# Characterizing centrality: Obsidian consumption, supra-regional connectivity, and social reproduction at the Early Bronze Age sanctuary of Keros (Cyclades, Greece)

**DOI:** 10.1371/journal.pone.0325218

**Published:** 2025-09-11

**Authors:** Tristan Carter, Rose Moir, Marina Milić, Colin Renfrew

**Affiliations:** 1 Department of Anthropology, McMaster University, Ontario, Canada; 2 School of Earth, Environment & Society, McMaster University, Ontario, Canada; 3 Irish Research Council, Ballsbridge, Ireland; 4 McDonald Institute for Archaeological Research, University of Cambridge, Cambridge, United Kingdom; Griffith University, AUSTRALIA

## Abstract

Early Bronze Age [EBA] Keros was a central place in the 3rd millennium cal BC Cycladic islands (Greece). Its material culture attests links with communities throughout the Aegean and beyond. This study uses obsidian sourcing to help reconstruct the socio-economic networks that coalesced at the site. Some 207 artifacts were elementally characterized using portable x-ray fluorescence spectroscopy [pXRF], the material coming from two ritual deposits in the Kavos area (n = 103), and the opposite islet settlement of Dhaskalio (n = 104). The results are consonant with the cosmopolitan character of Keros’ ceramic and metallurgical assemblages with not only the expected Melian sources of Dhemenegaki and Sta Nychia represented, but also handfuls of much rarer material from Giali A in the Dodecanese and East Göllü Dağ in central Anatolia. The study also provides further evidence for a Cycladic and Cretan preference for Sta Nychia raw materials in the EBA. A more complex picture of Melian obsidian consumption locally and regionally is then produced by integrating the sourcing data with the artifacts’ techno-typological and metrical attributes, which enables us to detail several EBA cultural traditions or ‘communities of practice’ across the Aegean region. The small quantities of Giali A and East Göllü Dağ obsidian are testimony to the supra-regional networks that coalesced at the site. Both raw materials likely circulated alongside the flow of Anatolian metals into the Aegean (including tin and gold), a network that introduced socially significant media and knowledge to Keros from as far east as the Indus. This congregation of people, resources, and technical know-how on Keros formed a key mode of social reproduction in Cycladic society with the mortuary and commemorative rituals on Kavos and the commensal gatherings on Dhaskalio comprising important spaces for the initiation, maintenance, and celebration of social relations.

## Introduction

**Fig 1:**
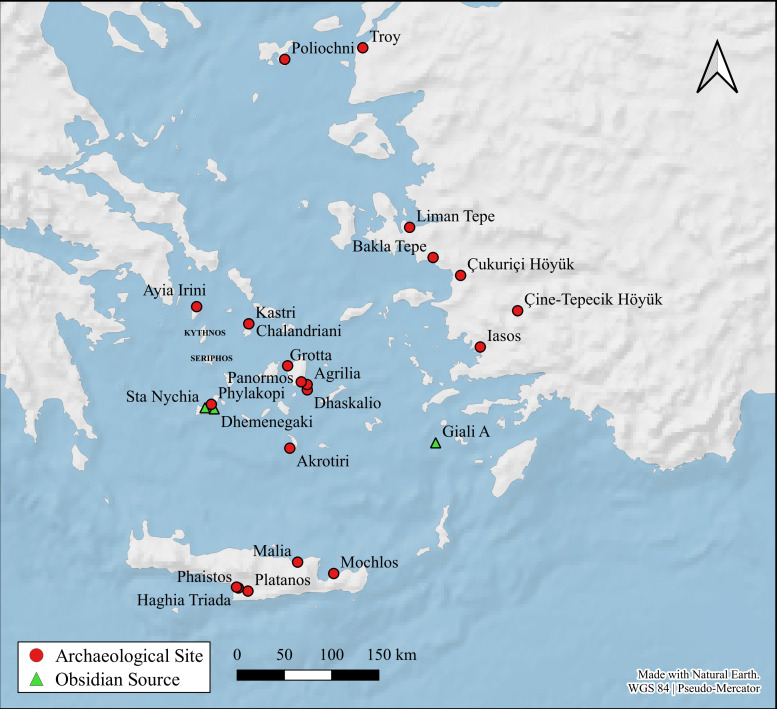
Keros and the obsidian sources considered in the study, plus distant sites mentioned in the text. Compiled in QGIS 3.16.3 using a basemap made from Natural Earth data by O. Crowdy.

**Fig 2:**
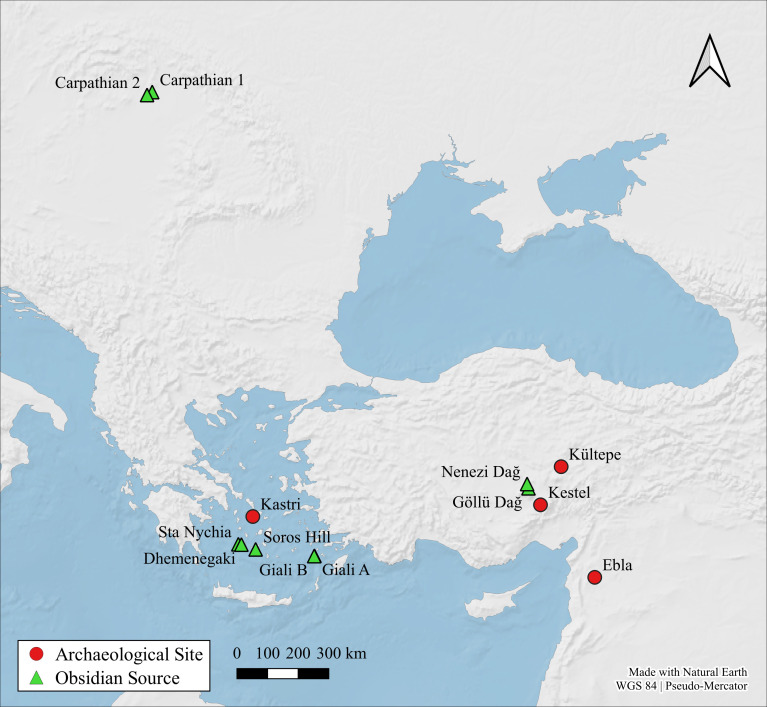
Map showing the main sites mentioned in the text. Compiled in QGIS 3.16.3 using a basemap made from Natural Earth data by O. Crowdy.

**Fig 3:**
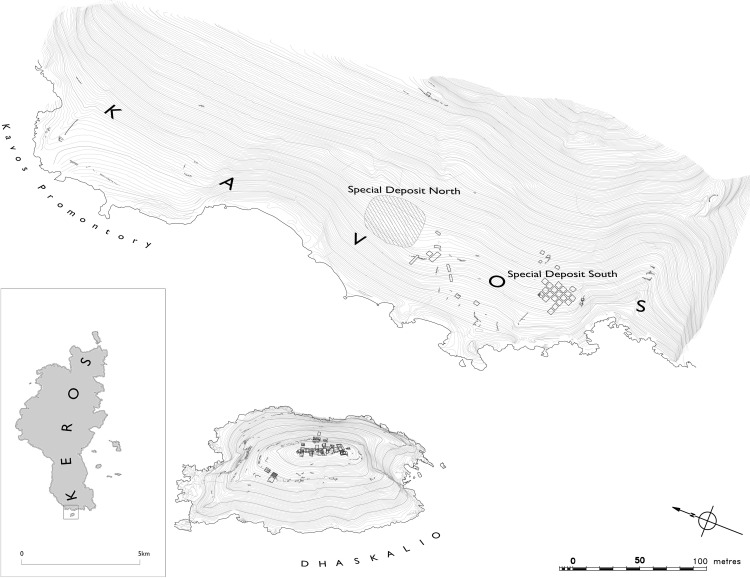
The western tip of Keros and the islet of Dhaskalio, showing the location of the looted Special Deposit North, plus the sanctuary at the Special Deposit South, and the settlement on Dhaskalio excavated between 2006−08. Reprinted from [2] under a CC BY license, with permission from Antiquity, original copyright 2012.

**Fig 4:**
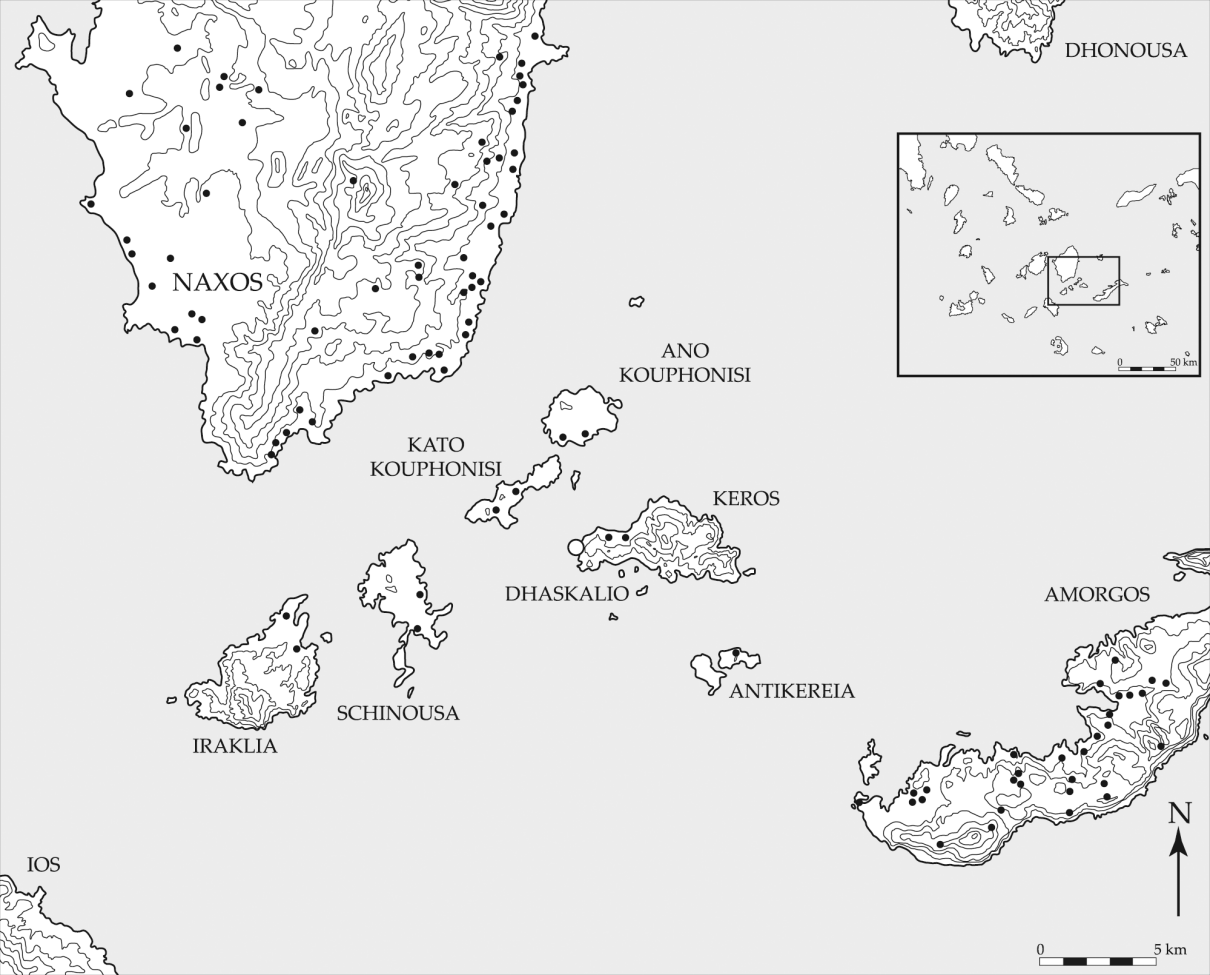
Map of the Small Cyclades (*Mikres Kyklades*) between Naxos and Amorgos, the black dots indicating Early Bronze Age sites. Reprinted from [7] under a CC BY license, with permission from the McDonald Institute for Archaeology, original copyright 2013.

Situated in the Cycladic archipelago of the southern Aegean Sea (Figs 1 and 2), the small island of Keros no longer has any perennial occupants yet some 5000 years ago in the Early Bronze Age [EBA] it was one of the Aegean’s most well-connected sites [1]. Its recent excavator has interpreted the masses of broken marble figurines at the so-called Kavos ‘Special Deposits’ on the island’s northwest coast (Fig 3) as the result of pan-Cycladic ritual gatherings, making Keros the “oldest maritime sanctuary” in the world [2]. The socio-economic centrality of Keros is even more remarkable when one considers the fact that the island has few natural resources of its own and limited agricultural potential. Indeed, the bulk of their building stone and foodstuffs were imported likely from southeast Naxos and nearby Kouphonisia respectively (Fig 4), while their pottery and tools – both lithic and metal – were procured from a much larger area [3–6].

This paper contributes to our understanding of the “complexity of the human practices and interconnections anchored at the site” [1] by reconstructing some of the socio-economic networks that coalesced at EBA Keros via an obsidian sourcing study. This analysis involved the elemental characterization of 207 obsidian artifacts from the 2006–08 excavations on Kavos (n = 103) and the settlement of Dhaskalio (n = 104), the latter material coming from three occupation phases that span around 500 years (from ~2750–2250 cal BCE) [2,8]. Obsidian was an off-island resource for the inhabitants of Keros with the visual inspection of these assemblages suggesting strongly that there was a greater variability of raw materials represented than was typical for a southern Aegean EBA site [9]. Methodologically, our analysis does not focus on raw material origin alone but provides the artifacts with a multi-faceted characterization through reference to their typo-technological and scalar attributes [10–14]. The results of such an approach enables us to map ‘communities of practice’ [15], i.e., shared traditions of consumption across space and time that are believed to reflect close connections between populations as likely articulated via marriages and other forms of deeply binding relationships [cf. 16,17]. The results of this study also contribute to the long-term exploitation history of the Aegean obsidian sources, which is a topic of research that extends back over 60 years to the first geochemical characterization studies of obsidian undertaken in a Eurasian context [18,19]. In contrast to the surrounding obsidian source regions of the Central and Western Mediterranean [20,21] and Anatolia to the east [22,23], the Aegean saw few characterization studies from the 1980’s until relatively recently with the Keros study forming part of a new suite of analyses [14, 24–29 *inter alia*] that have been largely facilitated by the introduction of portable x-ray fluorescence spectroscopy [30,31].

Aside from a handful of central European and Anatolian raw materials e.g., [32–34], it has long been held [e.g., 35] that most obsidian artifacts from Greek and western Turkish prehistoric sites were fashioned from Aegean source products, primarily those from Sta Nychia and Dhemenegaki on the western Cycladic island of Melos (Figs 1 and 2). While archaeometric analyses have shown this to be broadly true, there has been little consideration as to whether the two geo-spatially distinct Melian sources [36,37] had common histories of use in terms of when, how, and by whom they were exploited. While Sta Nychia and Dhemenegaki are only 12 km apart and have similar visual and knapping properties [38], they cannot be assumed to have been used or valued in the same way [39]. Indeed, in keeping with what we see at many other proximate obsidian sources, such as neighboring Göllü Dağ and Nenezi Dağ in central Anatolia e.g., [40] or the different outcrops of Monte Arci on Sardinia [e.g., 41], recent work is showing that Sta Nychia and Dhemenegaki were used differently over time [42,43]. This procurement bias is seen most clearly amongst Cycladic and Cretan Bronze Age populations [14,24,28,32]. Two of these studies are pertinent for contextualizing the Keros results (Figs 2 and 4) as they include data from several contemporary assemblages, including material from cemeteries on nearby Ano Kouphonisi and Naxos [28] and the settlement of Kastri on Syros in the northern Cyclades [14].

### Background to Keros

The significance of Keros as an EBA site first became apparent in the 1960’s when a wealth of marble anthropomorphic sculptures (‘figurines’) were dug up by looters at Kavos (Fig 3) on the island’s northeastern tip [44,45], the contents of the so-called ‘Keros hoard’ then being dispersed overseas via the illicit antiquities trade [46,47]. Once the looting became common knowledge, formal excavations were initiated by the Greek Archaeological Service [48] followed by an intensive pedestrian survey of Kavos in the late 1980’s [49]. The Cambridge Keros Project then undertook two phases of excavation on the island between 2006−08, and again from 2016−18 [1]. The artifacts analyzed in this paper derive from the 2006−08 campaign [9,50], though brief reference is made to Carter’s studies of the material from the 2016−18 excavation.

Through a combination of intensive fieldwork and anecdotal evidence, it is now believed that the looted area of ‘Special Deposit North’ on Kavos comprised both a cemetery, comparable in construction and wealth to that of Aplomata on northwest Naxos, and a focus of ritual dedicatory activity [2,51]. The excavations of 2006–08 revealed a second area of ritual activity, the ‘Special Deposit South’ (Fig 3), which produced a rich assemblage of 550 broken marble figurines, 2236 pieces of stone vessels, 53639 pottery sherds, and 3452 obsidian artifacts mainly in the form of fine pressure blades [52,53]. Studies indicate that the marble goods were deliberately broken prior to deposition, the act of fragmentation having likely occurred on other islands, before the material was carried overseas to the sanctuary at Kavos [2]. As detailed below, the inclusion of obsidian within the Special Deposit South formed an “integral part” of the rituals performed at this locale “conceptually analogous” to the deposition of the marble items, though some of these implements were manufactured on-site [50].

Two other areas on Kavos investigated in 2006−08 need to be mentioned briefly (Fig 3) as a few obsidian artifacts from Area A (n = 7) and the Middle Area (n = 11) were also included in this study. At the southern edge of the Special Deposit South is a small escarpment within which are three small rock-shelters used for burial, investigated as Kavos Area A [54]. The Middle Area of Kavos is situated between the two special deposits (Fig 3), the archaeology comprising a small oblong structure (the ‘Doumas House’) used by people involved in smelting metal ores plus other buildings in trench BA [55].

**Fig 5:**
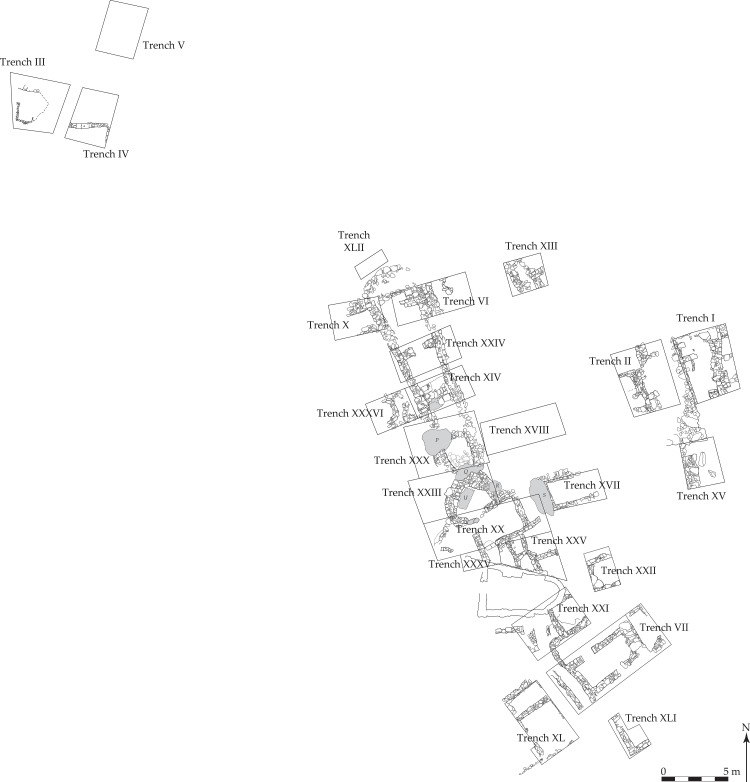
Plan of the trenches excavated at Dhaskalio, 2007−08. Reprinted from [57] under a CC BY license, with permission from the McDonald Institute for Archaeology, original copyright 2013.

Dhaskalio was a major EBA settlement part-contemporary with the two special deposits. Today it is an islet less than 100 m opposite Kavos (Fig 3), but during the EBA was joined to Keros by a peninsula [56]. Three occupation phases have been defined based upon ceramic typology and stratigraphy with radiocarbon dating producing an absolute chronology of: Phase A 2750–2550 cal. BCE; Phase B 2550–2400 cal. BCE, and Phase C 2400–2250 cal. BCE [2,8]. The steep sides of the islet meant that it was necessary to create terraces to support any construction, the buildings fashioned from marble that was imported in tonnes from Naxos some 10 km across the water [1]. The 2007−08 excavations on Dhaskalio focused on the summit area (Figs 3 and 5) with most structures and deposits relating to the Phase C occupation. A long and narrow structure (“the Hall”), plus a small enclosure, and courtyard formed a complex of non-domestic conceivably public character, while another building to the south was largely dedicated to storage [1]. A much smaller area of Phase B occupation was exposed with Phase A deposits fewer still.

Functionally, the lack of recognizable domestic spaces such as kitchens or middens led to the claim that Dhaskalio was established to periodically host large gatherings of visitors with few permanent residents, and most food being produced on neighboring islands [1]. Metal and obsidian working is not only attested throughout the site in each phase, but also at levels above and beyond anywhere else in the EBA Cyclades. While the former was focused on arsenical copper, there is also evidence for the processing of precious metals plus lead and bronze [1]. The origin of these resources is discussed further below in the context of our discussion of the obsidian sourcing results.

## Materials and methods

### The Keros obsidian assemblages

All necessary permits were obtained for the described study, which complied with all relevant regulations. Specifically, permission to work on the material detailed in this paper was provided by the Cycladic Ephorate of Antiquities – regional representative of the Hellenic Ministry of Culture. The artifacts were analyzed in a secure Ephorate repository in Chora on the island of Naxos where the Keros Project finds are stored.

The artifacts included in this study derive from four excavation areas with three datasets from Kavos and one from Dhaskalio. The former material mainly comes from the Special Deposit South with lesser quantities from Area A and the Middle Area. The flaked stone assemblages recovered from the 2006−08 Kavos and Dhaskalio excavations were comprised entirely of non-local raw materials with obsidian dominating (>98%) all four assemblages (Table 1). Dhaskalio produced the largest quantity of non-obsidian artifacts (n = 11, 0.8% of its total assemblage), mainly ready-made prismatic pressure blades of various colored cherts and red radiolarite (n = 7), five of which had glossed edges – a form of macroscopic use-wear produced by cutting silica-rich plants [58] suggesting that these implements were procured as sickle elements [9].

**Table 1:** The quantity of obsidian from the deposits detailed in the paper, and the proportion of those assemblages elementally characterized. FS = flaked stone; SDS = Special Deposit South (data from [9,50]).

Assemblage	Total FS	Obsidian (n.)	Obsidian (%)	Analysed (n.)	Analyzed (%)
Kavos SDS	3456	3452	99.9	85	2.5
Kavos Area A	125	124	99.2	7	5.6
Kavos Middle	315	315	100	11	3.5
Dhaskalio A	58	58	100	4	7
Dhaskalio B	504	502	99.6	36	7
Dhaskalio C	992	981	98.9	64	6.5

#### The Kavos Special Deposit South assemblage.

**Fig 6:**
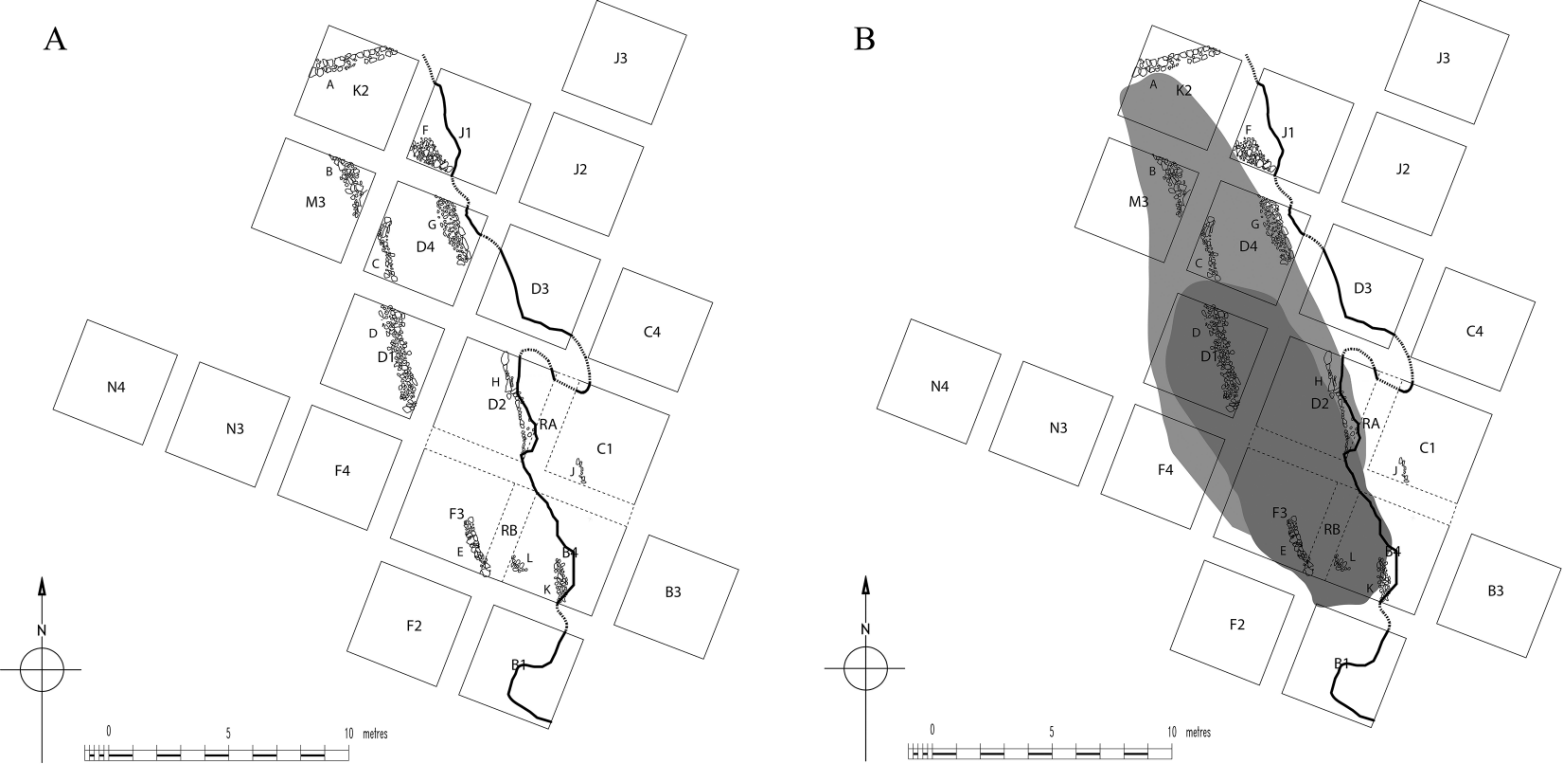
6a Stone features recorded in the Special Deposit South; 6b obsidian finds by excavation trench within the Special Deposit South. Reproduced from [59] under a CC BY license, with permission from the McDonald Institute for Archaeology, original copyright 2015.

The Special Deposit South was situated on a natural ledge on the hillside (15–25 masl) formed by an aeolianite formation that dropped a meter down to the limestone bedrock [53]. The deposit’s basal stratum comprised a paleosol with only a few artifacts, that was overlain by a layer representing the major phase of human activity at the site which included the construction of a few rough stone features (Fig 6a) running parallel to the aeolianite scarp [53]. Large quantities of material culture were recovered from around and between these linear features, though the exact sequence of events is difficult to reconstruct due to pit-digging being a part of the rituals performed here, actions that mixed the deposit [52]. This deposit, which included the topsoil, was eventually covered by a cairn of stones – the entire sequence embodying around 300 years of use. While obsidian artifacts were included in the Special Deposit South amidst the mass of broken marble objects, their distribution is not entirely the same with obsidian more evenly distributed (Fig 6b), the greatest concentrations on the aeolianite scarp whereas most other objects came from amongst the linear stone features [53]. Further distinctions can then be noted in the obsidian assemblage’s distribution namely: (a) obsidian was preferentially deposited in the earliest level (where it was possible to discern strata), (b) there is a notable concentration of pressure blades on the aeolianite shelf (where other objects are believed to have been placed/displayed), and (c) there is evidence for blade production occurring around the edges of the Special Deposit South [50,52].

Excavation of the Special Deposit South generated 3452 pieces of worked obsidian and four chert artifacts. The dominance of obsidian within a Cycladic EBA assemblage was entirely to be expected as was its techno-typological character, i.e., that it related to the manufacture of pressure blades [60,61]. Most of the obsidian had visual characteristics associated with raw materials from Melos (n = 3539/3542, 99.9%), i.e., pearl-grey to black, typically opaque with a few part-translucent and/or banded [19,62]. Visually distinguishing the products of the two Melian sources is difficult, even allowing for the fact that the more lustrous, translucent, and banded examples *tend* to come from Dhemenegaki rather than Sta Nychia [32], though such material is also known from Nenezi Dağ in central Anatolia [31]. With a very small amount of the latter source material having been documented in the EBA Aegean [29,63], it makes the need for a chemical characterization study even clearer.

**Fig 7:**
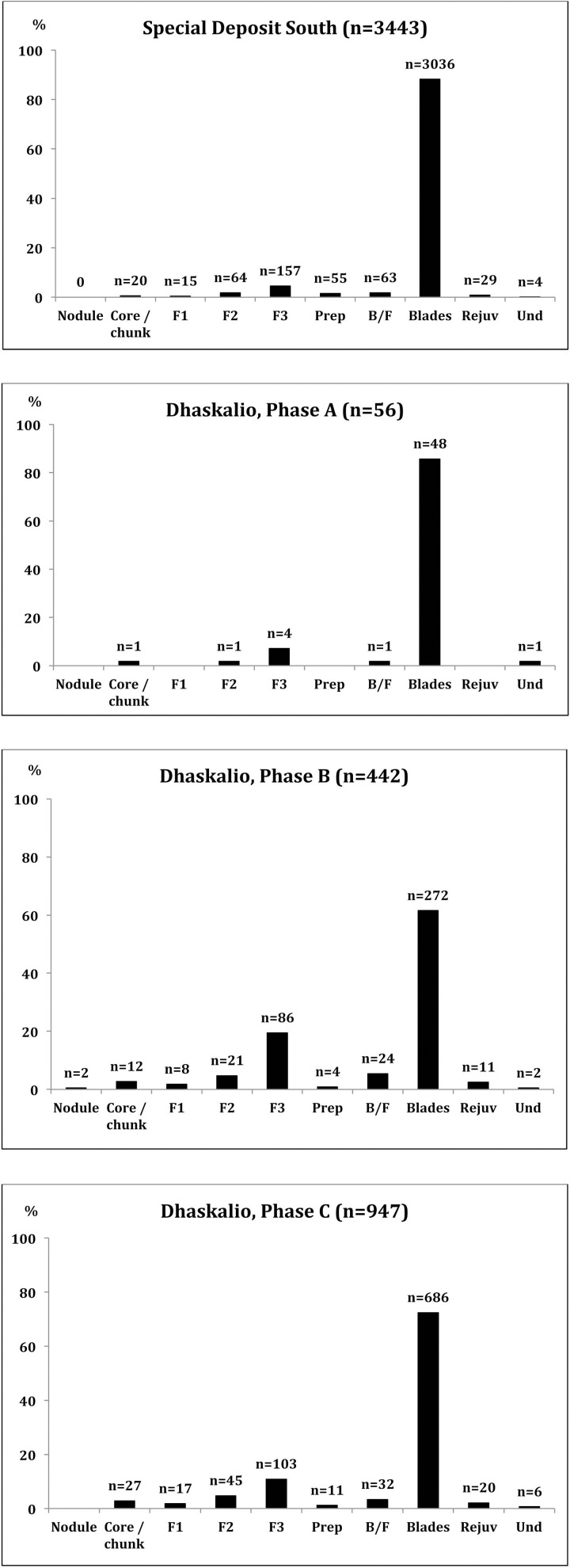
Techno-typological classes represented in the Kavos Special Deposit South, and Dhaskalio Phases A, B and C Melian obsidian assemblages, as determined by visual characterization (F1 = flake with 80–100% cortex; F2 = 5–80% cortex; F3 = 0–5% cortex; Prep = preparation piece; B/F = blade-like flake; Rejuv = rejuvenation; Und = undiagnostic). Reprinted from [9] under a CC BY license, with permission from the McDonald Institute for Archaeology, original copyright 2013.

**Fig 8:**
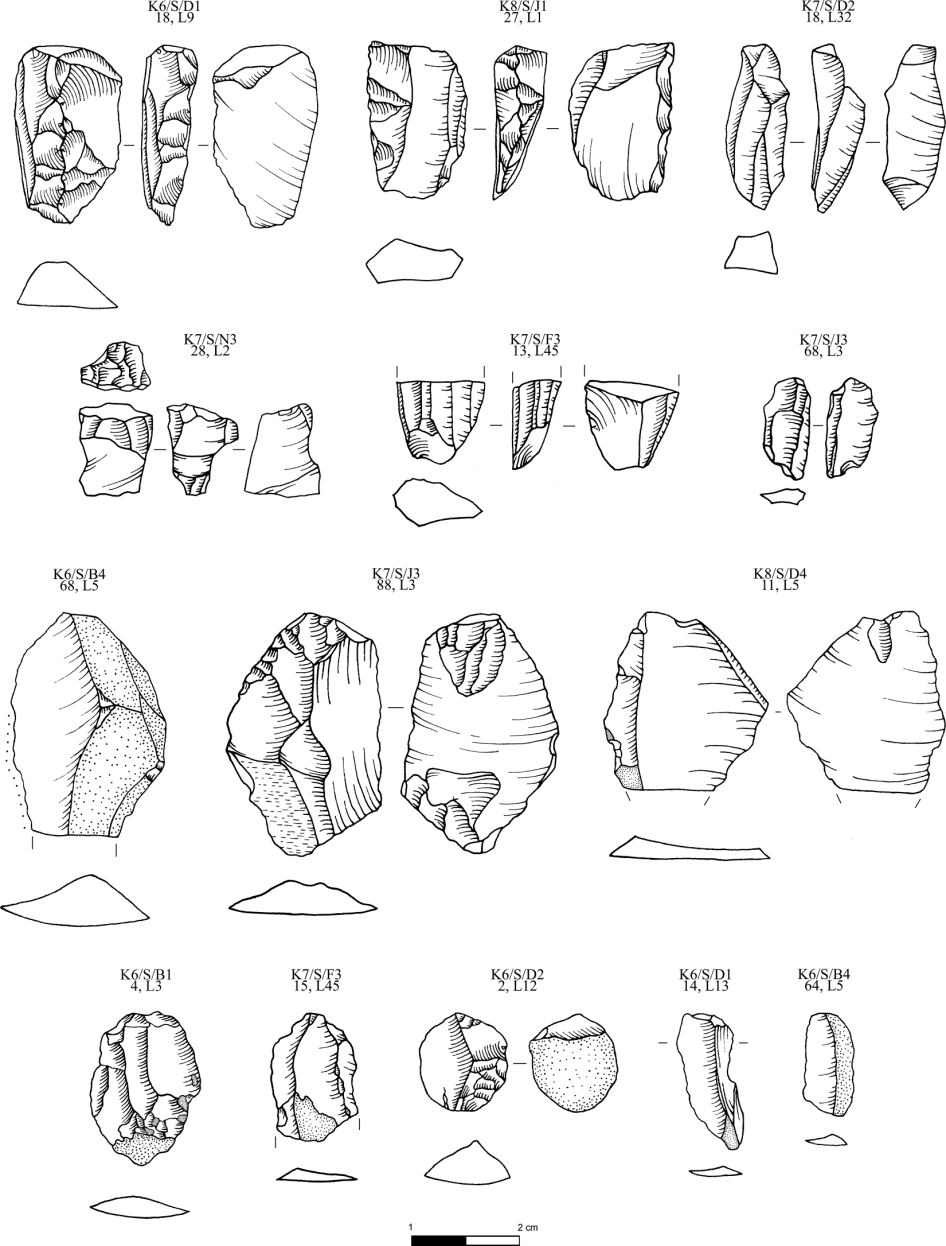
Obsidian pressure blade cores and cortical production and rejuvenation flakes from the Special Deposit South, Kavos analyzed in this study; all raw materials sourced to Sta Nychia. M. Milić. Original copyright with the authors.

**Fig 9:**
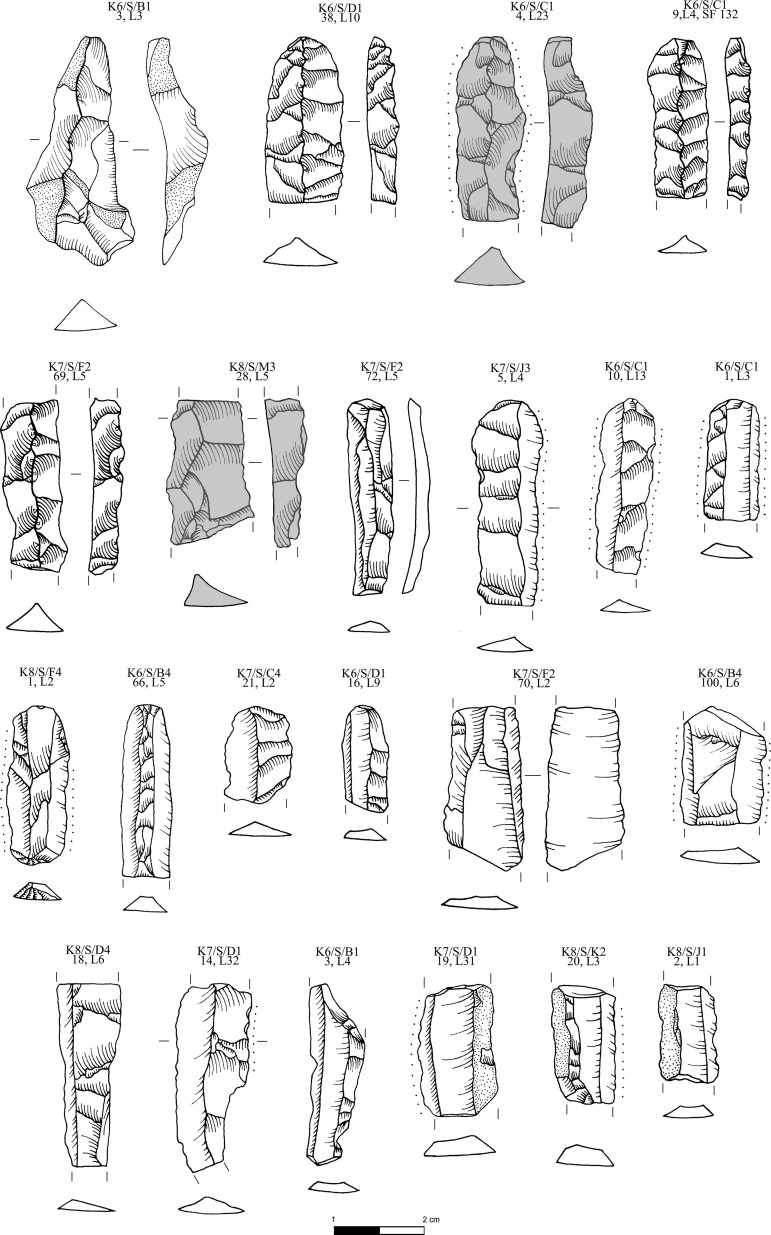
Obsidian initial (crested) and secondary series blades from the Special Deposit South, Kavos analyzed in this study; all raw materials sourced to Sta Nychia, except those highlighted in grey, from Dhemenegaki. M. Milić. Original copyright with the authors.

**Fig 10:**
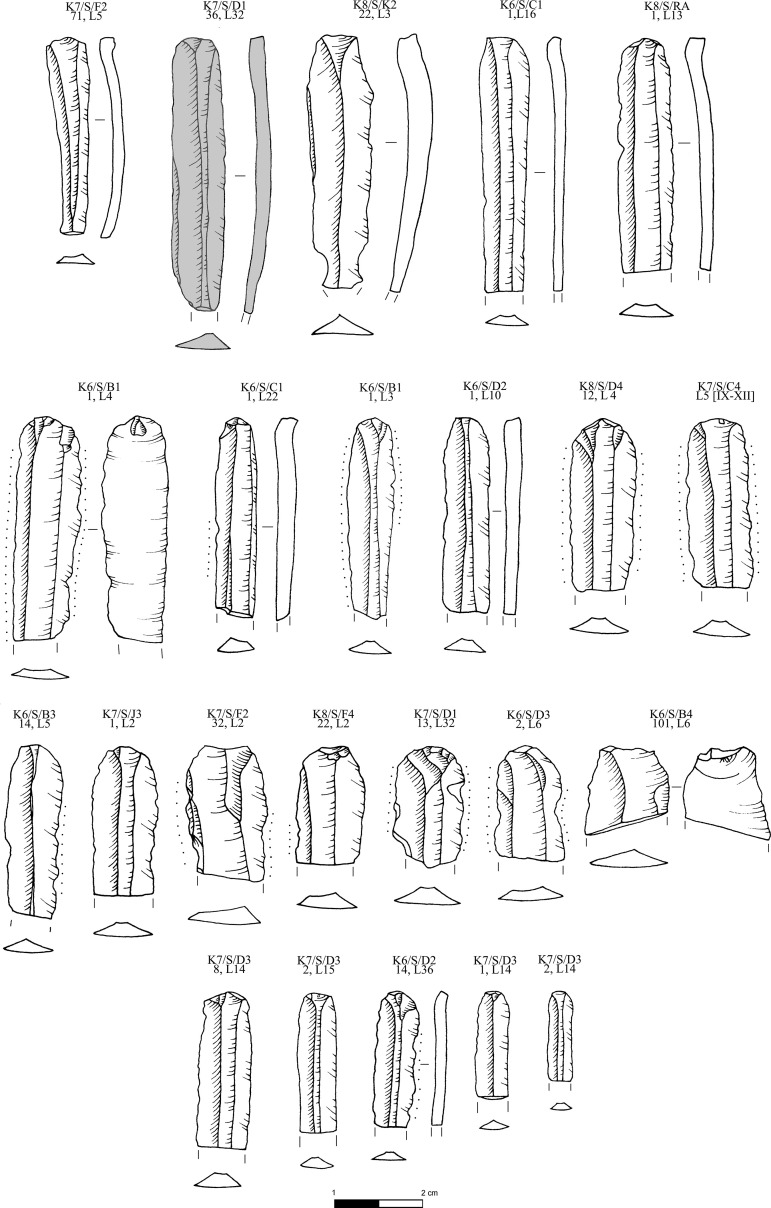
Obsidian prismatic pressure blades from the Special Deposit South, Kavos analyzed in this study; all raw materials sourced to Sta Nychia, except those highlighted in grey, from Dhemenegaki. M. Milić. Original copyright with the authors.

**Fig 11:**
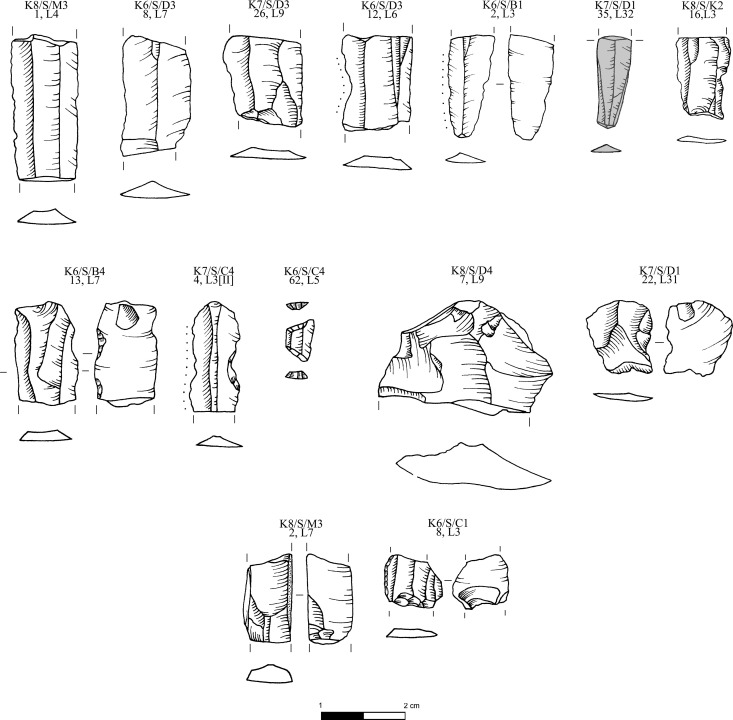
Obsidian prismatic pressure blades from the Special Deposit South, Kavos and core rejuvenation pieces analyzed in this study; all raw materials sourced to Sta Nychia, except those highlighted in grey, from Dhemenegaki. M. Milić. Original copyright with the authors.

Structurally the alleged Melian obsidian assemblage from Kavos is very homogenous (Figs 7–11) with 88% of the artifacts recorded as blades (n = 3036), a minimum of 938 implements originally based on the number of proximal segments represented. This contrasts with the material from Dhaskalio where blades constitute 62% and 72% of the (larger) Phase B and C datasets respectively (see below). Of further note is that the Melian obsidian blade assemblage from the Special Deposit South is skewed towards true prismatic end-products (95%) with initial or secondary series blades with cresting and/or cortex underrepresented (Fig 7), whereas in the Dhaskalio Phase B and C datasets end-products comprise only 88% and 86% of all blades respectively [50]. In sum, the consumption of Melian obsidian at the Special Deposit South was carefully structured with a clear preference for fine razor-sharp prismatic blades, a bias that was not unexpected given the character of the material recovered in the 1987 survey across Kavos [64]. That said, as noted above, there is evidence for a certain amount of blade production around the edges of the Special Deposit South with cores and an array of preparation and rejuvenation pieces (Fig 8) indicating that the ritual consumption of fine obsidian blades on Kavos comprised a mixture of implements made in the immediate vicinity plus quantities of material disinterred from off-island cemeteries (Figs 9–11). Ultimately, the techno-typological character of the Melian obsidian assemblage from the Special Deposit South is closely comparable to those recovered from funerary contexts throughout the EBA Cyclades [65,66]. It is thus suggested that these fine pressure blades from this part of Kavos were redeposited alongside other marble and ceramic grave goods disinterred from cemeteries throughout the archipelago or beyond (see below).

Finally, the Special Deposit South assemblage also included two chunks and a flake (all < 2.1 cm long) of a distinctive white spotted and lustrous obsidian, characteristics long associated with the Giali A source in the Dodecanese [19,67], ~ 138 km linear distance from Keros to the east-southeast (Fig 1).

#### The Kavos Area A assemblage.

The excavation of the rock-shelters and related deposits in Area A produced 125 flaked stone artifacts, 124 of which were visually characterized as Melian obsidian plus one flake of red chert. Most of the obsidian came from the trenches A1 (n = 76) and A2 (n = 37) that investigated inside and in front of Rock-shelter 1, respectively. The former assemblage was slightly more varied, including not only 45 fragmentary pressure blades (minimum of 17) but also a small quantity of production debris, with a core, chunk, an array of flakes (n = 20), and blade-like flakes (n = 9). The material from in front of Rock-shelter 1 was more homogenous and thus more typical of the finds one associates with Cycladic EBA grave goods with pressure blades dominating the assemblage (n = 32/37, five implements minimum) together with a few flakes.

A further 11 pieces of obsidian came from in front of Rock-shelter 1 (trench A3) all in the form of broken pressure blades – a minimum of four originally.

#### The Kavos Middle Area assemblage.

The 315 flaked stone artifacts from excavations in the Middle Area came from 13 trenches, just under a third of which came from trench AE (n = 102, 32%). The obsidian once again appeared to all be Melian, the blanks deriving from a pressure blade tradition (Fig 11) with the material likely having a varied use related to tasks associated with metalworking to ritual practices *inter alia*. While broken prismatic blades dominated (n = 211, 60 minimum originally), there was also some production debris with cores/chunks (n = 6), primary and secondary series blades (n = 21), flakes (including cortical material), and preparation and rejuvenation pieces.

The artifact ID for the Kavos material should be read as follows: K6/S/D1,18, L9 = Keros 2006, [Special Deposit] South, Trench D1, artifact ID 18, level 9, while K8/M/BA,1 L1 = Keros 2006, Middle, Trench BA, artifact ID 1, layer 1. If the initials ‘SF’ appear at the end of the artifact ID, it refers to the object being designated a three-dimensionally recorded ‘special find’. The Kavos artifacts included in the characterization study are detailed in Table 2, listed following their arrangement on Figs 8–11.

**Table 2:** The analyzed Kavos obsidian artifacts, by context, source, and blank type. (RS = Rock-shelter).

Artifact ID	Area	Source	Object	Figure
K6/S/D1,18 L9	SDS	Sta Nychia	Blade core – exhausted	Fig 8
K8/S/J1,27 L1	SDS	Sta Nychia	Blade core – exhausted	Fig 8
K7/S/D2,18 L32	SDS	Sta Nychia	Blade core – exhausted	Fig 8
K7/S/N3,28 L2	SDS	Sta Nychia	Blade core – exhausted	Fig 8
K6/S/F3,13 L45	SDS	Sta Nychia	Blade core – exhausted	Fig 8
K7/S/J3,68 L3	SDS	Sta Nychia	Blade core – exhausted	Fig 8
K6/S/B4,68 L5	SDS	Sta Nychia	Rejuvenation flake	Fig 8
K7/S/J3,88 L3	SDS	Sta Nychia	Rejuvenation flake – *pièce esquillée*	Fig 8
K8/S/D4,11 L5	SDS	Sta Nychia	Rejuvenation flake	Fig 8
K6/S/B1,4 L3	SDS	Sta Nychia	Rejuvenation flake	Fig 8
K7/S/F3,15 L45	SDS	Sta Nychia	Rejuvenation flake	Fig 8
K6/S/D2,2 L12	SDS	Sta Nychia	Cortical flake – *pièce esquillée*	Fig 8
K6/S/D1,14 L13	SDS	Sta Nychia	Cortical blade-like flake	Fig 8
K6/S/B4,64 L5	SDS	Sta Nychia	Cortical blade-like flake	Fig 8
K6/S/B1,3 L3	SDS	Sta Nychia	Crested blade	Fig 9
K6/S/D1,38 L10	SDS	Sta Nychia	Crested blade	Fig 9
K6/S/C1,4 L23	SDS	Dhemenegaki	Crested blade – notched	Fig 9
K6/S/C1,9 L4 SF 132	SDS	Sta Nychia	Crested blade	Fig 9
K7/S/F2,69 L5	SDS	Sta Nychia	Crested blade	Fig 9
K8/S/M3,28 L5	SDS	Dhemenegaki	Crested blade	Fig 9
K7/S/F2,72 L5	SDS	Sta Nychia	Secondary series blade	Fig 9
K7/S/J3,5 L4	SDS	Sta Nychia	Secondary series blade	Fig 9
K6/S/C1,1 L13	SDS	Sta Nychia	Secondary series blade	Fig 9
K6/S/C1,1 L3	SDS	Sta Nychia	Secondary series blade	Fig 9
K8/S/F4,1 L2	SDS	Sta Nychia	Secondary series blade	Fig 9
K6/S/B4,66 L5	SDS	Sta Nychia	Secondary series blade	Fig 9
K7/S/C4,21 L2	SDS	Sta Nychia	Secondary series blade	Fig 9
K6/S/D1,16 L9	SDS	Sta Nychia	Secondary series blade	Fig 9
K7/S/F2,70 L2	SDS	Sta Nychia	Secondary series blade	Fig 9
K6/S/B4,100 L6	SDS	Sta Nychia	Secondary series blade	Fig 9
K8/S/D4,18 L6	SDS	Sta Nychia	Secondary series blade	Fig 9
K7/S/D1,14 L32	SDS	Sta Nychia	Secondary series blade	Fig 9
K6/S/B1,3 L4	SDS	Sta Nychia	Secondary series blade	Fig 9
K7/S/D1,19 L31	SDS	Sta Nychia	Secondary series blade	Fig 9
K8/S/K2,20 L3	SDS	Sta Nychia	Secondary series blade	Fig 9
K8/S/J1,2 L1	SDS	Sta Nychia	Secondary series blade	Fig 9
K7/S/F2,71 L5	SDS	Sta Nychia	Prismatic blade	Fig 10
K7/S/D1,36 L32	SDS	Dhemenegaki	Prismatic blade	Fig 10
K8/S/K2,22 L3	SDS	Sta Nychia	Prismatic blade	Fig 10
K6/S/C1,1 L16	SDS	Sta Nychia	Prismatic blade	Fig 10
K8/S/RA,1 L13	SDS	Sta Nychia	Prismatic blade	Fig 10
K6/S/B1,1 L4	SDS	Sta Nychia	Prismatic blade	Fig 10
K6/S/C1,1 L22	SDS	Sta Nychia	Prismatic blade	Fig 10
K6/S/B1,1 L3	SDS	Sta Nychia	Prismatic blade	Fig 10
K6/S/D2,1 L10	SDS	Sta Nychia	Prismatic blade	Fig 10
K8/S/D4,12 L4	SDS	Sta Nychia	Prismatic blade	Fig 10
K7/S/C4 L5 [IX-XII]	SDS	Sta Nychia	Prismatic blade	Fig 10
K6/S/B3,14 L5	SDS	Sta Nychia	Prismatic blade	Fig 10
K7/S/J3,1 L2	SDS	Sta Nychia	Prismatic blade	Fig 10
K7/S/F2,32 L2	SDS	Sta Nychia	Prismatic blade	Fig 10
K8/S/F4,22 L2	SDS	Sta Nychia	Prismatic blade	Fig 10
K7/S/D1,13 L32	SDS	Sta Nychia	Prismatic blade	Fig 10
K6/S/D3,2 L6	SDS	Sta Nychia	Prismatic blade	Fig 10
K6/S/B4,101 L6	SDS	Sta Nychia	Prismatic blade	Fig 10
K7/S/D3,8 L14	SDS	Sta Nychia	Prismatic blade	Fig 10
K7/S/D3,2 L15	SDS	Sta Nychia	Prismatic blade	Fig 10
K6/S/D2,14 L36	SDS	Sta Nychia	Prismatic blade	Fig 10
K7/S/D3,1 L14	SDS	Sta Nychia	Prismatic blade	Fig 10
K7/S/D3,2 L14	SDS	Sta Nychia	Prismatic blade	Fig 10
K8/S/M3,1 L4	SDS	Sta Nychia	Prismatic blade	Fig 11
K6/S/D3,8 L7	SDS	Sta Nychia	Prismatic blade	Fig 11
K6/S/D3,26 L9	SDS	Sta Nychia	Prismatic blade	Fig 11
K6/S/D3,12 L6	SDS	Sta Nychia	Prismatic blade	Fig 11
K6/S/B1,2 L3	SDS	Sta Nychia	Prismatic blade (bipolar scars)	Fig 11
K7/S/D1,35 L32	SDS	Sta Nychia	Prismatic blade	Fig 11
K8/S/K2,16 L3	SDS	Sta Nychia	Prismatic blade	Fig 11
K6/S/B1,2 L4	SDS	Sta Nychia	Prismatic blade – notched	Fig 11
K6/S/B4,13 L7	SDS	Sta Nychia	Prismatic blade – denticulated	Fig 11
K7/S/C4,4 L3 [II]	SDS	Sta Nychia	Prismatic blade – notched	Fig 11
K6/S/C4,62 L5	SDS	Sta Nychia	Prismatic blade – trapeze	Fig 11
K8/S/D4,7 L9	SDS	Sta Nychia	Rejuvenation flake	Fig 11
K7/S/D1,22 L31	SDS	Sta Nychia	Rejuvenation flake	Fig 11
K8/S/M3,2 L7	SDS	Sta Nychia	Rejuvenation flake	Fig 11
K6/S/C1,8 L3	SDS	Sta Nychia	Rejuvenation flake	Fig 11
K7/M/AB,2 L8	Middle	Sta Nychia	Prismatic blade	Fig 11
K7/M/AB,24 L3	Middle	Sta Nychia	Prismatic blade	Fig 11
K7/M/AC,4 L3	Middle	Sta Nychia	Prismatic blade	Fig 11
K7/M/AB,6 L8	Middle	Sta Nychia	Prismatic blade/ rejuvenation piece	Fig 11
K8/M/AE,21 L2	Middle	Sta Nychia	Rejuvenation flake (core tablet)	Fig 11
K6/S/A1, L7	SDS	Sta Nychia	Prismatic blade	not shown
K6/S/A1, L20	SDS	Sta Nychia	Prismatic blade	not shown
K6/S/A1, L21	SDS	Sta Nychia	Prismatic blade	not shown
K6/S/A1, L24	SDS	Sta Nychia	Cortical flake	not shown
K6/S/A1, L28	SDS	Sta Nychia	Prismatic blade	not shown
K6/S/B4,64 L5	SDS	Sta Nychia	Cortical blade-like flake	not shown
K6/S/C1,8 L3	SDS	Sta Nychia	Secondary series blade	not shown
K7/S/C4,1 L5	SDS	Giali A	Chunk (1.98 × 1.96 × 1.06 cm)	not shown
K8/S/D4,12 L4	SDS	Sta Nychia	Prismatic blade	not shown
K8/S/K2,1 L7	SDS	Giali A	Chunk (2.09 × 1.53 × 0.63 cm)	not shown
K8/S/K2,1 L11	SDS	Giali A	Flake (1.79 × 0.73 × 0.5 cm)	not shown
K8/S/A2, L1	Area A/ RS1	Sta Nychia	Prismatic blade	not shown
K8/S/A2, L2	Area A/ RS1	Sta Nychia	Prismatic blade	not shown
K8/S/A2, L4	Area A/ RS1	Sta Nychia	Prismatic blade	not shown
K8/S/A3, L1	Area A/ RS2	Sta Nychia	Prismatic blade	not shown
K8/S/A3, L2	Area A/ RS2	Sta Nychia	Prismatic blade	not shown
K8/S/A3, L7	Area A/ RS2	Sta Nychia	Prismatic blade	not shown
K8/S/A3, L11	Area A/ RS2	Sta Nychia	Prismatic blade	not shown
K8/M/AD, L2	Middle	Sta Nychia	Blade core	not shown
K8/M/AD, L4	Middle	Sta Nychia	Prismatic blade	not shown
K8/M/AE, L3	Middle	Sta Nychia	Prismatic blade	not shown
K8/M/AE, L4	Middle	Sta Nychia	Prismatic blade	not shown
K8/M/AE, L12	Middle	Sta Nychia	Prismatic blade	not shown
K8/M/BA,1 L1	Middle	Dhemenegaki	Blade-like flake	not shown

#### The Dhaskalio assemblage.

The 2007−08 excavation of the Dhaskalio settlement generated 1554 chipped-stone artifacts, an assemblage once again dominated by obsidian (n = 1541, 99.2% [Table 1]). Of the three occupation horizons, the earliest – Phase A – was least well represented and produced only 67 obsidian artifacts, 4% of the assemblage, with Phase B generating 495 items (32% of the total, with two chert pieces), while Phase C deposits were dominant and richest in lithics with 992 artifacts (64% of the whole), all except nine of which were made of obsidian [9].

**Fig 12:**
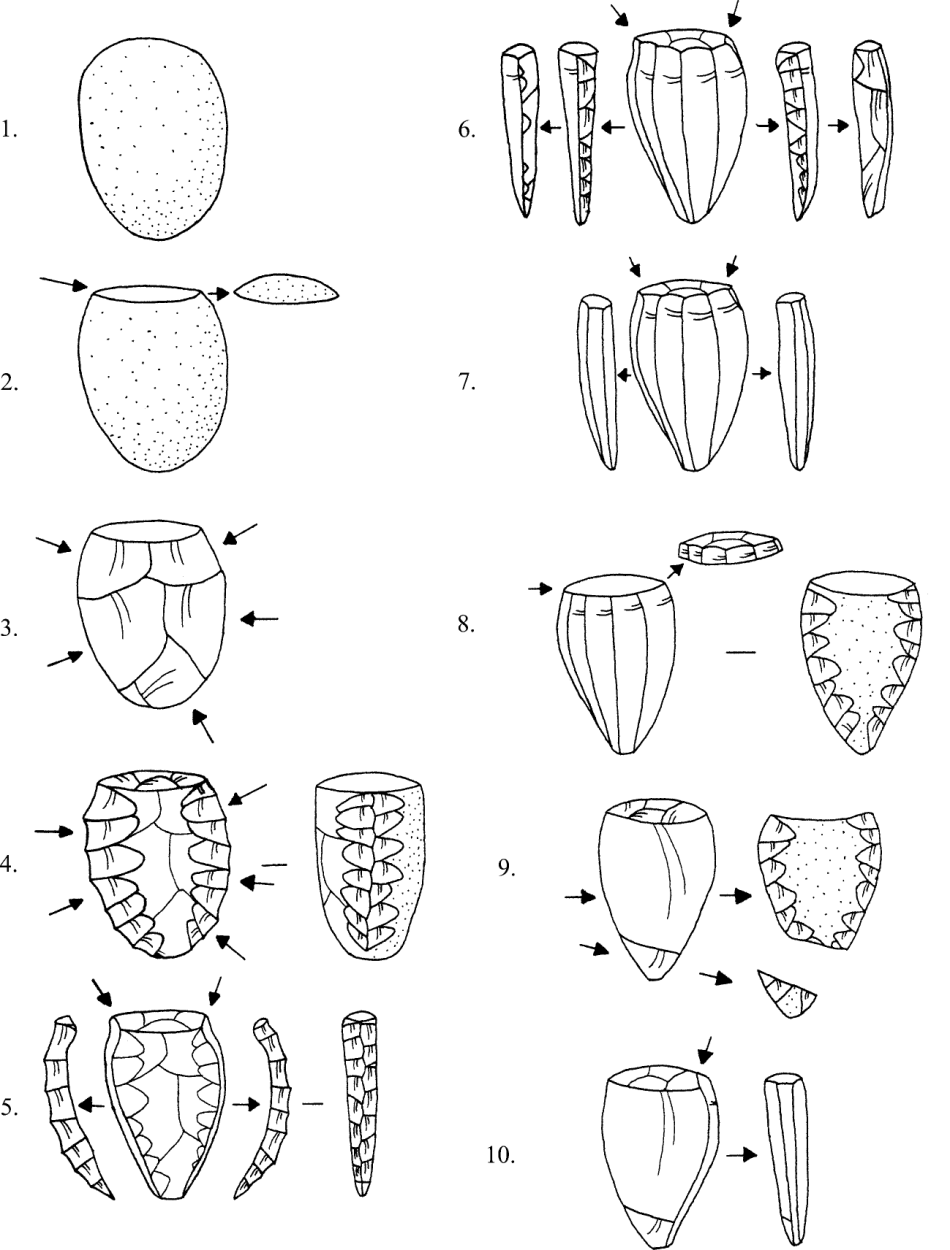
General reconstruction of Early Bronze Age obsidian blade-core preparation and reduction sequence in the Cyclades. L. Labriola. Original copyright with the authors.

**Fig 13:**
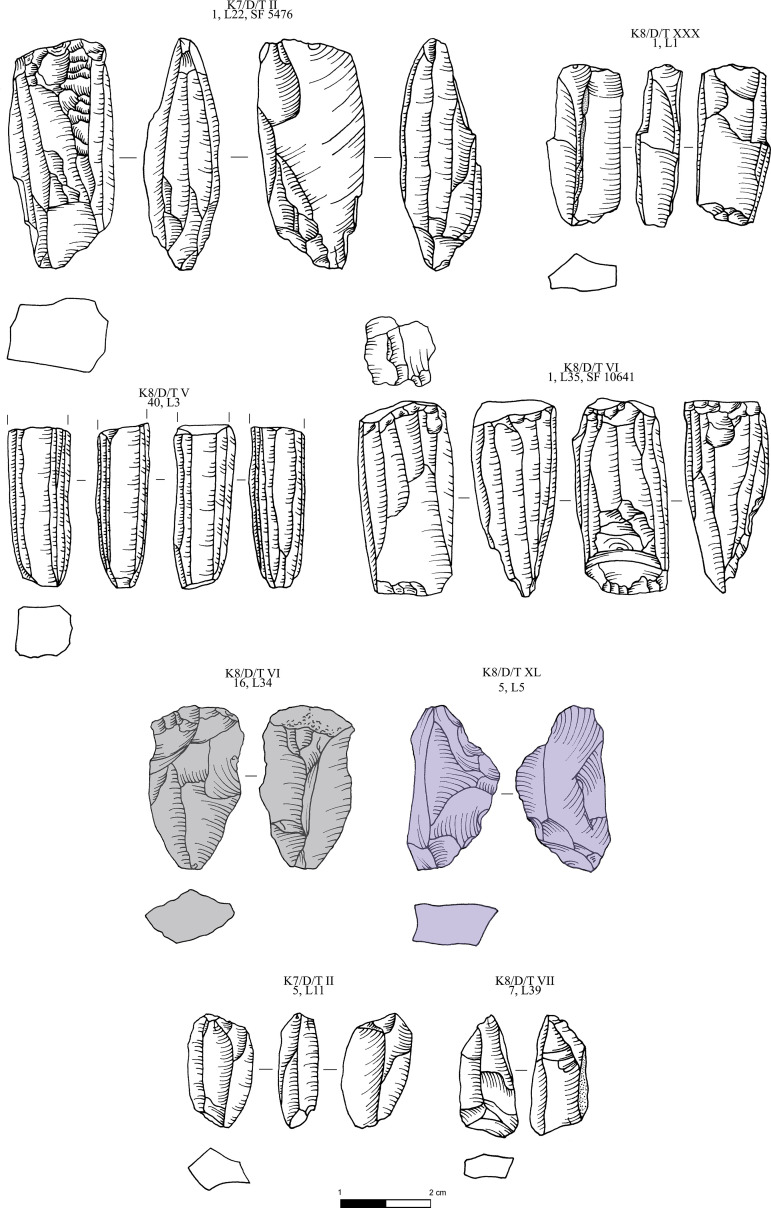
Obsidian pressure blade cores from Dhaskalio analyzed in this study; all raw materials sourced to Sta Nychia, except those highlighted in grey, from Dhemenegaki, and those in purple, from East Göllü Dağ. M. Milić. Original copyright with the authors.

**Fig 14:**
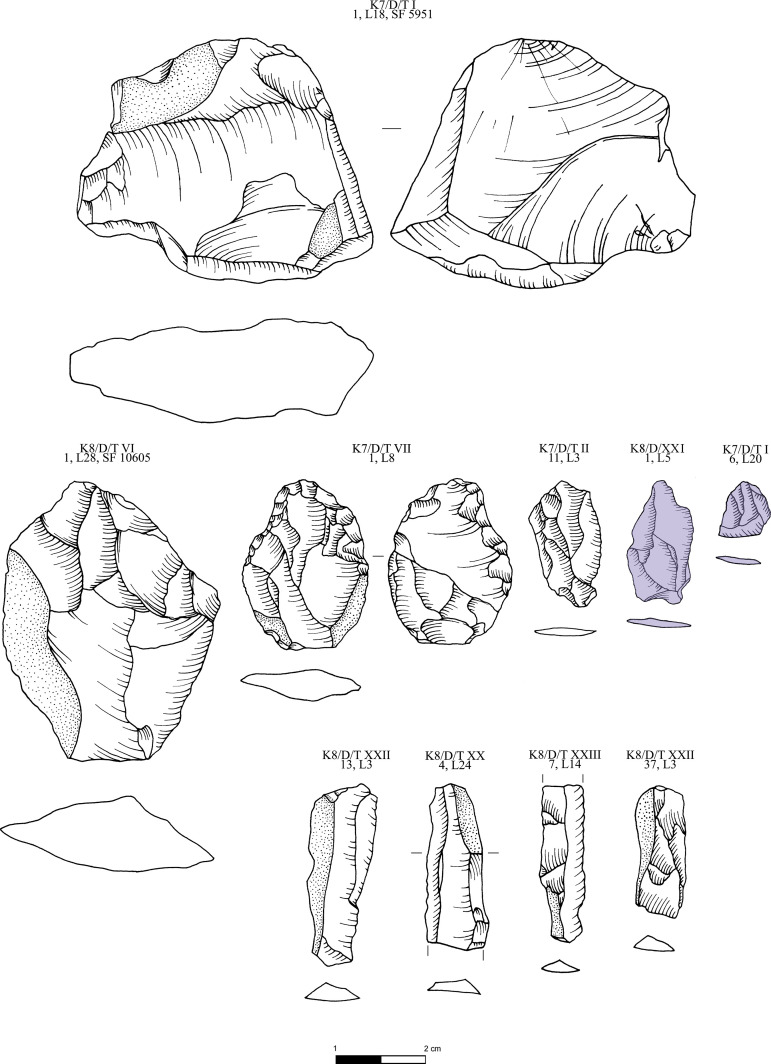
Obsidian cores, flakes and blades from Dhaskalio analyzed in this study; all raw materials sourced to Sta Nychia, except those highlighted in grey, from Dhemenegaki, and those in purple, from East Göllü Dağ. M. Milić. Original copyright with the authors.

**Fig 15:**
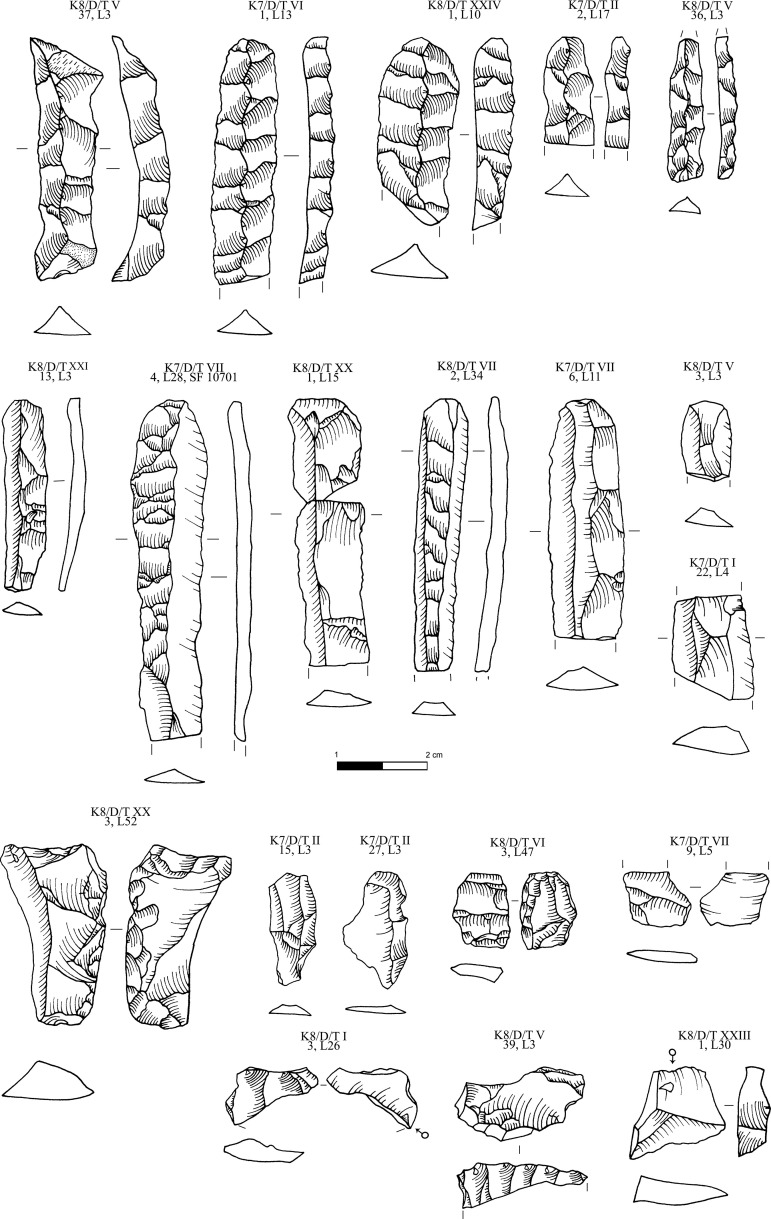
Obsidian pressure blades and rejuvenation flakes from Dhaskalio analyzed in this study; all raw materials sourced to Sta Nychia. M. Milić. Original copyright with the authors.

**Fig 16:**
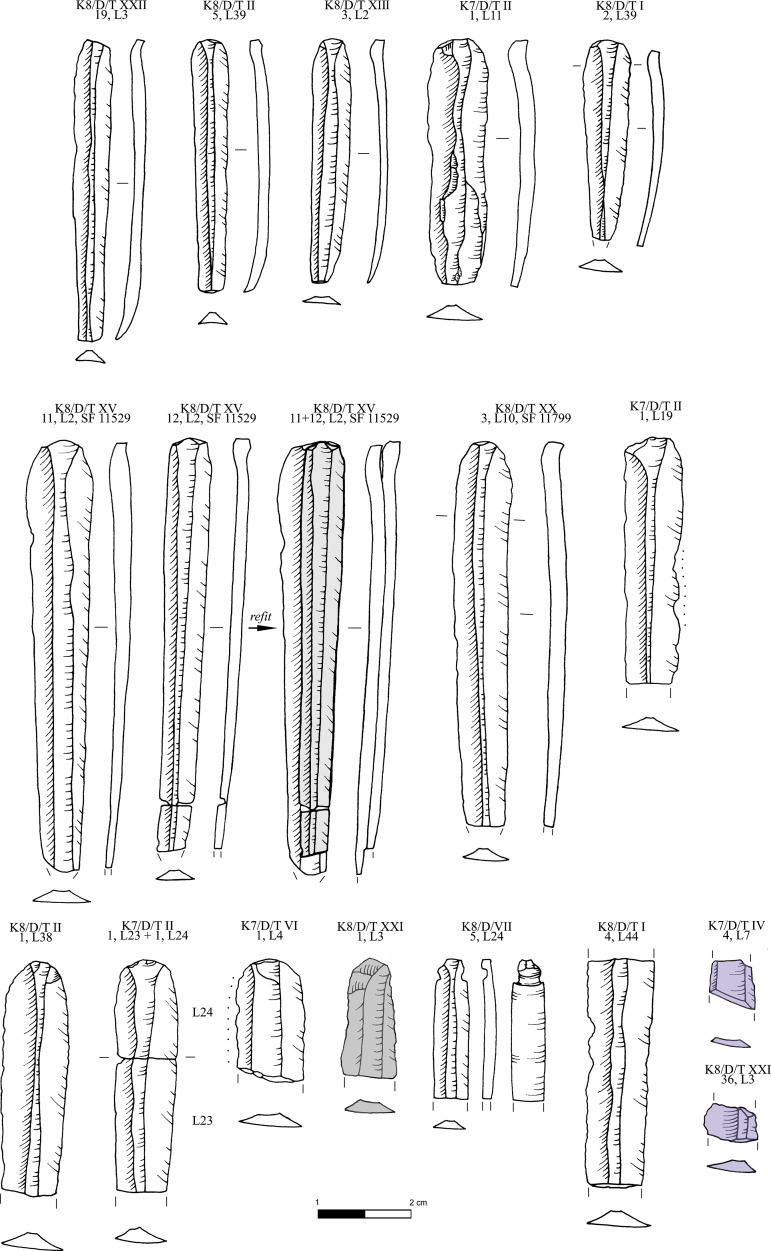
Obsidian prismatic pressure blades from Dhaskalio; all raw materials sourced to Sta Nychia, except those highlighted in grey, from Dhemenegaki, and those in purple, from East Göllü Dağ. M. Milić. Original copyright with the authors.

While most of the obsidian again appeared to be Melian (99.6%, n = 1535), this assemblage was structurally more heterogeneous than that from Kavos containing a much large proportion of cores, waste flakes, and technical pieces (Fig 7), which is evidence of on-site production of pressure flaked blades (Fig 12). The relative lack of cortical debris indicates that Melian obsidian was brought to Dhaskalio having been largely decorticated and shaped elsewhere conceivably at the sources to test the nodules’ quality and reduce transport weight [62]. On site the Dhaskalio obsidian workers undertook the blade core’s final shaping and preparation, employing a series of technical choices that were common to Cycladic populations at this time [14,68]. Firstly, a flake was removed from one end of the nodule to create a working platform, then the knapping surface was further prepared by the removal of smaller flakes to provide facets for the tip of the pressure-flaking tool, the subsequent blades thus often having dihedral platforms on removal (Fig 12:2–3). Before any blades could be removed, it was necessary to carefully flake at least one artificial ridge (crest) down the core’s face – a feature that provided a path of least resistance for the fracture wave to travel along when initiated by the application of pressure at the junction of platform and crest (Fig 12:4–5). After the removal of the crested blade the subsequent blades have remnant cresting scars on one side of their dorsal surfaces, after which the knapper attains the full rhythm of production (*plein débitage*), generating prismatic blades with parallel margins and ridges and trapezoidal cross-section (Fig 12:6–7). Significant quantities (n = 874) of these prismatic blades were recovered throughout the excavation. Using the number of proximal sections to estimate the minimum quantity of end-products represented, there were at least 17 blades originally from the Phase A deposits (total 44), 77 from Phase B (total 238), and 188 from Phase C (total 592). A representative selection of the obsidian cores, technical pieces, and prismatic blades from Dhaskalio Phases A-C are detailed in Figs 13–16.

**Fig 17:**
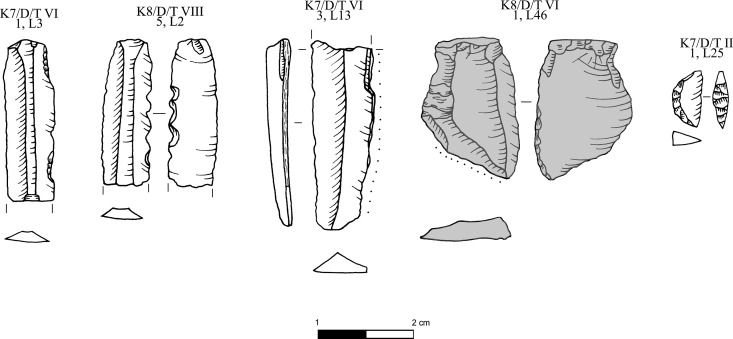
Obsidian retouched prismatic pressure blades and flakes from Dhaskalio; all raw materials sourced to Sta Nychia, except those highlighted in grey, from Dhemenegaki. M. Milić. Original copyright with the authors.

In keeping with EBA Cycladic obsidian consumption traditions, most of these razor-sharp pressure blades were used unmodified with only 3% of the prismatic end-products (n = 26/874) having been retouched, the most common type being notched pieces including one true denticulate plus a few pieces with simple linear modification, backed pieces, and a single trapeze (Fig 17). Such modified pieces, not least the trapeze, find direct parallels from the Kastri assemblage on Syros which should be contemporary with Dhaskalio Phase B [14]. A more specialized piece with a polished edge from a Phase C context (Fig 17 [K7/D/VI,3 L13]) is likely to have been used for stone carving, potentially the incision of lines in a marble figurine or vessel [cf. 69].

The Melian obsidian assemblage from each phase also includes blanks deriving from episodes of core rejuvenation which variously involved flaking a new platform (sometimes on the opposing end of the nucleus leading to bidirectional blade scars) or by opening a new face for reduction by removing a posterior crest and working what was originally the back of the core (Fig 12:8–9). Once again, these technical strategies are well-attested in other EBA Cycladic assemblages and to an extent the larger southern Aegean throughout the Bronze Age [e.g., 14,24,68,70,71 *inter alia*]. The relative proportion and absolute quantity of the various techno-typological blanks documented in the Dhaskalio Melian obsidian assemblage by phase is detailed in Fig 7.

**Fig 18:**
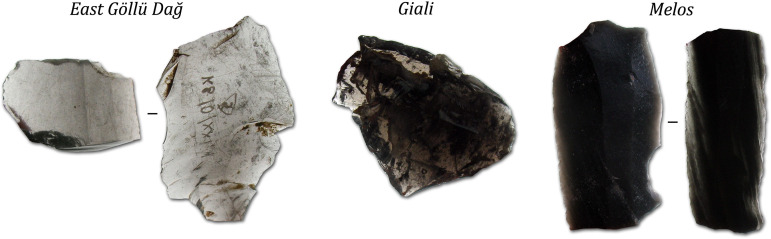
Various obsidian types from the Keros excavations. Reprinted from [9] under a CC BY license, with permission from the McDonald Institute for Archaeology, original copyright 2013.

The original study of the Dhaskalio assemblage also claimed that it contained at least one, and maybe as many as three, pieces of the white-spotted Giali A obsidian from the Dodecanese – the material comprising small (≤ 2.1 cm) non-cortical flakes, one from Phase B and two from Phase C [9]. It was also suggested that there were five artifacts made of obsidian from central Anatolia, specifically the East Göllü Dağ ‘source’ of southern Cappadocia some 900 km to the east of Keros as-the-crow-flies (Fig 1). This claim was based upon the raw material’s lustrous surface, translucency, and purple/grey hue (Fig 18), attributes the authors recognized as characteristic of this source material based upon their experience of undertaking combined visual and elemental characterization of Göllü Dağ products from Neolithic Çatalhöyük where it was represented in abundance [9,10,40,72,73 *inter alia*]. As with the Melian obsidian, those artifacts made of alleged Cappadocian raw materials derived from a pressure blade industry with two exhausted cores, two blade fragments, and a rejuvenation flake from the face of a nucleus (Figs 13–14 and 16). One of the blades came from a Phase B context and the other four objects from Phase C deposits (Table 3).

**Table 3:** The analyzed Dhaskalio obsidian artifacts, by context, phase, source and blank type.

Artifact ID	Phase	Source	Object	Figure
K7/D/T II,1 L22, SF 5476	B	Sta Nychia	Blade core	Fig 13
K8/D/T XXX,1 L1	C	Sta Nychia	Blade core	Fig 13
K8/D/T V,40 L3	B	Sta Nychia	Blade core	Fig 13
K8/D/T VI,1 L35, SF 10641	C	Sta Nychia	Blade core	Fig 13
K8/D/T VI,16 L34	C	Dhemenegaki	Blade core(?) – exhausted	Fig 13
K8/D/T XL,5 L5	C	East Göllü Dağ	Blade core(?) – exhausted	Fig 13
K7/D/T II,5 L11	B	Sta Nychia	Blade core – exhausted	Fig 13
K8/D/T VII,7 L39	C	Sta Nychia	Blade core – exhausted	Fig 13
K7/D/T I,1 L18, SF 5951	B	Sta Nychia	Nodule	Fig 14
K8/D/T VI,1 L28, SF 10605	C	Sta Nychia	Core fragment	Fig 14
K7/D/T VII,1 L8	C	Sta Nychia	Blade core – *pièce esquillée*	Fig 14
K7/D/T II,11 L3	B	Sta Nychia	Blade core(?) – exhausted	Fig 14
K8/D/T XXI,1 L5	C	East Göllü Dağ	Blade core(?) – exhausted	Fig 14
K7/D/T I,6 L20	B	East Göllü Dağ	Rejuvenation flake	Fig 14
K8/D/T XXII,13 L3	C	Sta Nychia	Secondary series blade	Fig 14
K8/D/T XX,4 L24	C	Sta Nychia	Secondary series blade	Fig 14
K8/D/T XXIII,7 L14	C	Sta Nychia	Secondary series blade	Fig 14
K8/D/T XXII,37 L3	C	Sta Nychia	Secondary series blade	Fig 14
K8/D/T V,37 L3	B	Sta Nychia	Crested blade	Fig 15
K7/D/T VI,1 L13	C	Sta Nychia	Crested blade	Fig 15
K8/D/T XXIV,1 L10	C	Sta Nychia	Crested blade	Fig 15
K7/D/T II,2 L17	B	Sta Nychia	Crested blade	Fig 15
K8/D/T V,36 L3	B	Sta Nychia	Crested blade	Fig 15
K8/D/T XXI,13 L3	C	Sta Nychia	Secondary series blade	Fig 15
K8/D/T VII,4 L28 SF10701	C	Sta Nychia	Secondary series blade	Fig 15
K8/D/T XX,1 L15	C	Sta Nychia	Secondary series blade	Fig 15
K8/D/T VII,21 L34	C	Sta Nychia	Secondary series blade	Fig 15
K7/D/T VII,16 L11	C	Sta Nychia	Secondary series blade	Fig 15
K8/D/T V,3 L3	B	Sta Nychia	Secondary series blade	Fig 15
K7/D/T I,22 L4	B	Sta Nychia	Secondary series blade	Fig 15
K8/D/T XX,3 L52	C	Sta Nychia	Blade core – *pièce esquillée*	Fig 15
K7/D/T II,15 L3	B	Sta Nychia	Rejuvenation flake	Fig 15
K7/D/T II,27 L3	B	Sta Nychia	Rejuvenation flake	Fig 15
K8/D/T VI,3 L47	C	Sta Nychia	Rejuvenation flake	Fig 15
K7/D/T VII,9 L5	C	Sta Nychia	Rejuvenation flake	Fig 15
K8/D/T I,3 L26	B	Sta Nychia	Rejuvenation flake	Fig 15
K8/D/T V,39 L3	B	Sta Nychia	Rejuvenation flake – core tablet	Fig 15
K8/D/T XXIII,1 L30	C	Sta Nychia	Rejuvenation flake – core tablet	Fig 15
K8/D/T XXII,19 L3	C	Sta Nychia	Prismatic blade	Fig 16
K8/D/T II,5 L39	A	Sta Nychia	Prismatic blade	Fig 16
K8/D/T XIII,3 L2	C	Sta Nychia	Prismatic blade	Fig 16
K7/D/T II, L11	B	Sta Nychia	Prismatic blade	Fig 16
K8/D/T I,2 L39	B	Sta Nychia	Prismatic blade	Fig 16
K8/D/T XV,11 L2 SF 11529	C	Sta Nychia	Prismatic blade	Fig 16
K8/D/T XV,12 L2 SF 11529	C	Sta Nychia	Prismatic blade	Fig 16
K8/D/T XX,3 L10 SF 11799	C	Sta Nychia	Prismatic blade	Fig 16
K7/D/T II,1 L19	B	Sta Nychia	Prismatic blade	Fig 16
K8/D/T II,1 L38	A	Sta Nychia	Prismatic blade	Fig 16
K7/D/T II,1 L23	A	Sta Nychia	Prismatic blade	Fig 16
K7/D/T VI,1 L4	C	Sta Nychia	Prismatic blade	Fig 16
K8/D/T XXI,1 L3	C	Dhemenegaki	Prismatic blade	Fig 16
K8/D/T VII,5 L24	C	Sta Nychia	Prismatic blade	Fig 16
K8/D/T I,4 L44	B	Sta Nychia	Prismatic blade	Fig 16
K8/D/T IV4, L7	B	East Göllü Dağ	Prismatic blade	Fig 16
K8/D/T XXI,36, L3	C	East Göllü Dağ	Prismatic blade	Fig 16
K7/D/T VI,1 L3	C	Sta Nychia	Prismatic blade – notched	Fig 17
K8/D/T VIII,5 L2	C	Sta Nychia	Prismatic blade – denticulated	Fig 17
K7/D/T VI,3 L13	C	Sta Nychia	Prismatic blade – polished edge	Fig 17
K8/D/T VI,1 L46	C	Dhemenegaki	Blade-like flake – retouched	Fig 17
K7/D/T II,1 L25	A	Sta Nychia	Prismatic blade – trapeze	Fig 17
K7/D/T I,1 L6	B	Dhemenegaki	Crested blade	not shown
K7/D/T I,1, L15	B	Sta Nychia	Prismatic blade	not shown
K7/D/T I, 1 L16	B	Sta Nychia	Prismatic blade	not shown
K8/D/T I, 1 L22	B	Sta Nychia	Prismatic blade	not shown
K7/D/T I, 1 L23, SF 10113	B	Sta Nychia	Cortical flake	not shown
K8/D/T I,1 L35	B	Sta Nychia	Prismatic blade	not shown
K8/D/T II,1 L9	B	Sta Nychia	Secondary series blade	not shown
K7/D/T II,1 L10	B	Sta Nychia	Prismatic blade	not shown
K7/D/T II,2 L10	B	Sta Nychia	Non-cortical flake	not shown
K7/D/T II,5 L11	B	Sta Nychia	Nodule	not shown
K7/D/T II,1 L13	B	Sta Nychia	Prismatic blade	not shown
K7/D/T II,1 L16	B	Sta Nychia	Non-cortical flake	not shown
K7/D/T II,1 L29	B	Sta Nychia	Prismatic blade	not shown
K7/D/T II,1 L30	B	Sta Nychia	Prismatic blade	not shown
K8/D/T II,1 L50	B	Sta Nychia	Secondary series blade	not shown
K7/D/T II,1 L51	B	Sta Nychia	Prismatic blade	not shown
K7/D/T VI,3 L12	C	Giali A	Non-cortical flake	not shown
K7/D/T VI,1 L20	C	Sta Nychia	Cortical flake	not shown
K8/D/T VI,1 L43	C	Sta Nychia	Cortical flake	not shown
K8/D/T VI,1 L49	C	Sta Nychia	Blade core – exhausted	not shown
K8/D/T VI,1 L50	C	Sta Nychia	Cortical flake	not shown
K7/D/T VII,7 L11	C	Sta Nychia	Prismatic blade	not shown
K8/D/T VII,21 L34	C	Sta Nychia	Secondary series blade	not shown
K8/D/T XIII, 2 L8	C	Giali A	Non-cortical flake	not shown
K8/D/T XXIV,1 L2	C	Sta Nychia	Prismatic blade	not shown
K8/D/T XXIV,1 L3	C	Sta Nychia	Prismatic blade	not shown
K8/D/T XXIV,2 L3	C	Sta Nychia	Prismatic blade	not shown
K8/D/T XXIV,1 L7	C	Sta Nychia	Prismatic blade	not shown
K8/D/T XXIV,2 L8	C	Dhemenegaki	Prismatic blade	not shown
K/D/T XXIV,1 L13	C	Sta Nychia	Prismatic blade	not shown
K8/D/T XVII,1 L1	C	Sta Nychia	Prismatic blade	not shown
K8/D/T XVII,1 L2	C	Sta Nychia	Prismatic blade	not shown
K8/D/T XVII,11 L2	C	Sta Nychia	Non-cortical flake	not shown
K8/D/T XVII,18 L2	C	Dhemenegaki	Cortical flake	not shown
K8/D/T XVII,4 L5	C	Sta Nychia	Prismatic blade	not shown
K8/D/T XVII,5 L5	C	Sta Nychia	Prismatic blade	not shown
K8/D/T XX,1 L8	C	Sta Nychia	Prismatic blade	not shown
K8/D/T XX,1 L30	C	Sta Nychia	Secondary series blade	not shown
K8/D/T XX,1 L32	C	Sta Nychia	Prismatic blade	not shown
K8/D/T XX,1 L33	C	Sta Nychia	Prismatic blade	not shown
K8/D/T XX,1 L53	C	Sta Nychia	Prismatic blade	not shown
K8/D/T XXI,13 L3	C	Sta Nychia	Secondary series blade	not shown
K8/D/T XXII,13 L3	C	Sta Nychia	Secondary series blade	not shown
K8/D/T XXII,37 L3	C	Sta Nychia	Secondary series blade	not shown

The artifact ID for the Dhaskalio material should be read as follows: K8/D/T VII,4 L28 SF10701 = Keros 2008, Dhaskalio, Trench VII, artifact ID 4, level 1. The Dhaskalio artifacts characterized in this study are detailed in Table 3 following their arrangement on Figs 13–17.

### Sampling strategy

The material selected for elemental characterization were primarily those artifacts illustrated in the original Dhaskalio and Kavos publications – the logic being that these pieces had been originally selected to represent the technical traditions, reduction sequences, and modified implements embodied within these assemblages (Figs 8–11 and 13–17). While these drawings included the five artifacts allegedly produced from Cappadocian obsidian, they did not include the flakes believed to be from Giali A as they were small and techno-typologically indistinct flakes, though one piece was photographed (Fig 18). The sourcing study did, however, involve all the alleged Giali A material (five from Kavos and one-to-three from Dhaskalio) plus one blade-like flake from the Kavos Middle Area excavation whose translucency and banding suggested that it might have been made of Nenezi Dağ obsidian (artifact K8/M/BA,1 L1). The total number of artifacts analyzed was 207, of which 103 came from Kavos (85 from the Special Deposit South, seven from Area A, and 11 from the Middle Area) and 104 from Dhaskalio. While this is one of the largest obsidian datasets to be elementally characterized in an Aegean context it remains a small sample of the total artifacts from the 2006–08 excavations, representing only 2.5% of the Special Deposit South material and between 6.5–7% of the Dhaskalio datasets (Table 1).

### Research questions

This study had several research questions in mind. The most basic was to clarify which source materials were represented in the Keros assemblage, as visually it is very difficult to discriminate the Melian raw materials and it was uncertain as to whether a handful of translucent pieces from Dhaskalio were made of Giali A and/or Göllü Dağ obsidian. As with any obsidian sourcing study, determining these artifacts’ raw material origins forms a means to a larger set of ends e.g., [74–77]. In this case, we aimed to use the results of the analyses to (1) reconstruct the socio-economic networks that coalesced at Keros (2) map distinct cultural traditions of obsidian consumption within the EBA Aegean, and (3) contribute to a long-term history of Aegean obsidian source exploitation.

The first research question relates to the long-acknowledged fact that EBA Keros represented an extraordinary site within its larger Cycladic/southern Aegean context. Over 30 years ago Broodbank [78,79] categorized Dhaskalio-Kavos as a ‘trader site’, i.e., one of a handful of Cycladic communities (Fig 2) demographically capable of producing and crewing the longboats that were of such great socio-economic significance to these islanders. While subsequent fieldwork has served to muddy the waters somewhat, it remains clear that certain communities stand out in terms of their size and connectivity including Ayia Irini on Kea, Chalandriani-Kastri on Syros, Palati-Grotta on Naxos, Skarkos on Ios, and likely Phylakopi on Melos and Akrotiri on Thera [3,80]. Another key characteristic of these sites is that they were centers of metalworking, obsidian pressure blade production, and the manufacture of technically complex and artistically elaborate ceramic vessels – the idea being that preferential access to skilled craftworkers and their goods formed a key form of socio-economic capital in EBA Cycladic society [see also 60,65]. Within this nexus of competing island factions, Keros stands apart in terms of its diversity and geographic reach embodied within its rich material culture assemblages; for example, the ceramic assemblage includes vessels procured from throughout the Cyclades, the Saronic Gulf, and the Greek mainland with a few pieces from Crete and western Anatolia [3,6]. Is the obsidian assemblage similarly heterogeneous compared to other EBA sites in the region and can we talk of common exchange routes for pots, metals, and obsidian or are these networks distinct in social and geographic organization and involve different value regimes?

The second research question relates to the increasing evidence for cultural regionalism in the EBA Aegean that crosscuts and subverts the long-standing [81] concepts of Cycladic, Minoan (Cretan), and Helladic (mainland) cultural spheres [see 79,82–86]. Answering this question involves the mapping of common crafting traditions as evidenced by the raw material, technological, and typological choices made by these EBA obsidian workers. The mapping of these communities of practice requires a multi-faceted approach to artifact characterization, one that melds raw material source, technical specifics, measurements, and typology [e.g., 10,11,14,24,32,87 *inter alia*].

The third aim of this study is to contribute to an understanding of Aegean obsidian source exploitation over the long-term. Melian obsidian is of particular interest here, being the dominant regional sources whose raw materials were exploited from the Upper Palaeolithic to the end of the Bronze Age [24,42] and was accessed by communities as far west as the Adriatic, as far south as Crete, as far east as western Anatolia, and as far north as Greek Macedonia [21,34,43,88]. Pertinent to this study is the relatively recent appreciation that southern Aegean Bronze Age populations clearly preferred Sta Nychia obsidian [14,24,28,32].

### Elemental characterization using pXRF

The elemental characterization of the 194 artifacts from Keros was achieved using a Bruker Tracer III-SD portable X-ray fluorescence spectrometer [pXRF], a well-established technique for obsidian sourcing studies in the Aegean [14,25,26,30,31,89]. The method is also attractive due to it being non-destructive, rapid, accurate, and generating reproducible data [90,91] which benefits anyone working in a region where cultural heritage regulations make it difficult to move artifacts from museum holdings to archaeometric laboratories.

The pXRF generates X-rays using a miniaturized X-ray tube with a Rh target and a maximum voltage of 40 kV, equipped with a silicon drift detector with a resolution of 145 eV. It has an analytical window of 10 mm in diameter with an X-ray beam of 5 × 7 mm, making it suitable for the analysis of smaller artifacts that otherwise could be problematic for analyses using a desktop X-ray fluorescence spectrometer [cf. 43,92]. Using 40 kV voltage and 38 μA anode current with the green filter in place we measured for the concentrations (in ppm) of 10 elements: Mn, Fe, Zn, Ga, Th, Rb, Sr, Y, Zr, and Nb.

**Fig 19:**
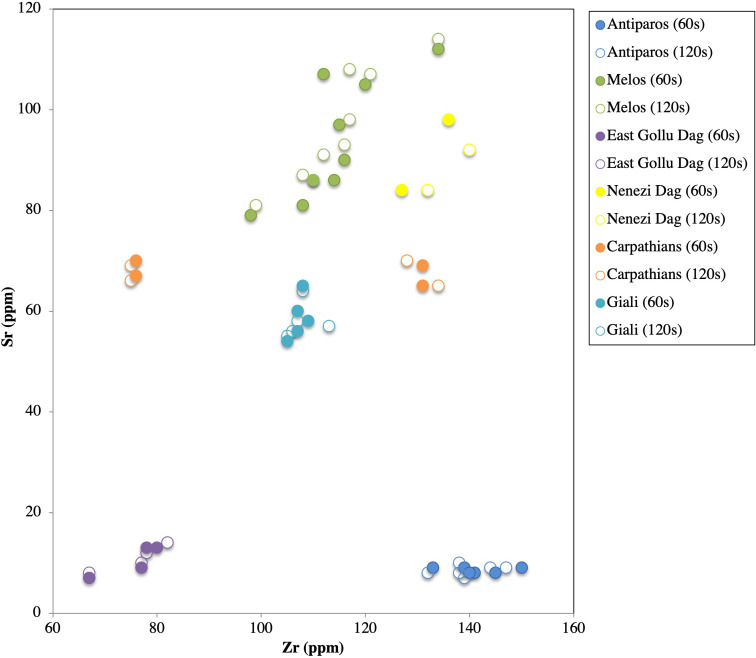
Bivariate contents plot of Zr vs. Sr for geological samples from the Aegean, Carpathians, and main central Anatolian obsidian sources considered in this study, detailing results of being analyzed by the pXRF for 60 and 120 seconds. R. Moir. Original copyright with the authors.

The artifacts having first been cleaned and air dried were placed over the detector window on their flattest and most even surface possible (usually the ventral) with 165 artifacts analyzed for 120 seconds each (including all the Kavos material), while 42 artifacts from Dhaskalio were measured for 60 seconds each. The latter protocol was employed towards the end of the study not only due to time restrictions but also to determine whether the difference in analytical time generated conflicting results. It was demonstrated that there was little difference between 120s and 60s in terms of discriminating the obsidian sources (Fig 19) [see also 93,94]. To ensure machine calibration and accuracy the USGS standard RGM-2 standard was analyzed every day before, periodically during, and after artifact analysis.

The Keros obsidian was then sourced by comparing the artifacts’ elemental profiles with those of geological samples of known origin generated under the same analytical conditions (Fig 19). The number of geological samples per source included in the study was sub-optimal due to time constraints and that the process involved transporting the source material from Canada to Greece. Samples from each of the Aegean sources (Fig 1) were included in the study plus those raw materials from the Carpathians (central Europe) and Cappadocia (central Anatolia) that has previously been documented in Aegean prehistoric assemblages [31,32,34,63,95]. This comprised geological samples from: Soros Hill (n = 5), Antiparos [96,97], Sta Nychia (n = 5) and Dhemenegaki (n = 3), Melos [36,37,97,98], Giali A (n = 3), Giali B (n = 2) [67,97], the ‘Carpathian 1’ (n = 2) and ‘Carpathian 2’ (n = 2] sources of eastern Slovakia, and north-east Hungary, respectively [99], plus East Göllü Dağ (n = 4) and Nenezi Dağ (n = 2) in southern Cappadocia [97,100].

To source the artifacts’ raw materials we initially focused upon the trace elements Rb, Sr, and Zr as these have been successfully used in contents and ratio plots to discriminate Aegean, Carpathian, and Cappadocian source products using XRF techniques [12,14,25,26,31,42,43 *inter alia*]. Subsequently, the major element Fe was considered as this has a discriminatory power for the Melian sources when using pXRF [14,30,31].

## Results

### Sourcing the Kavos ‘Special Deposit South’ assemblage

**Fig 20:**
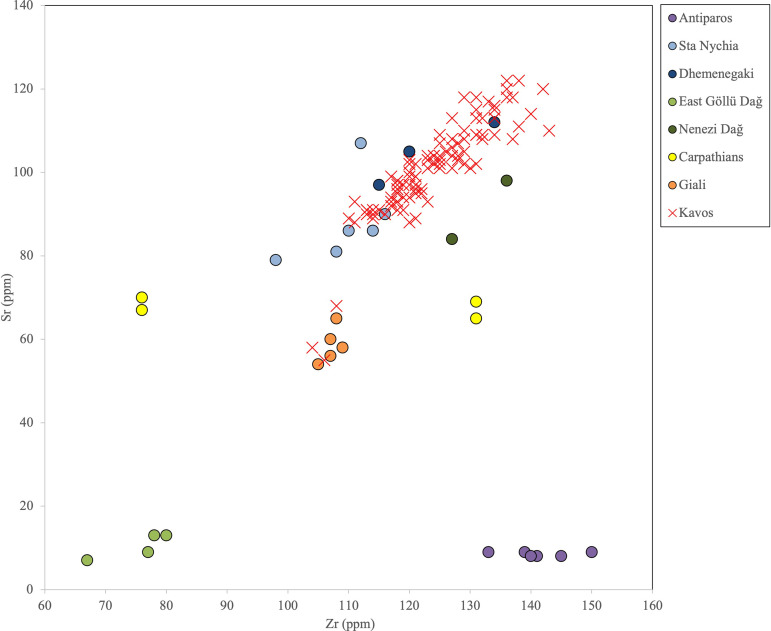
Bivariate contents plot of Zr vs. Sr for geological samples from the Aegean, Carpathians, and main central Anatolian obsidian sources, plus the 103 Kavos artifacts. R. Moir. Original copyright with the authors.

**Fig 21:**
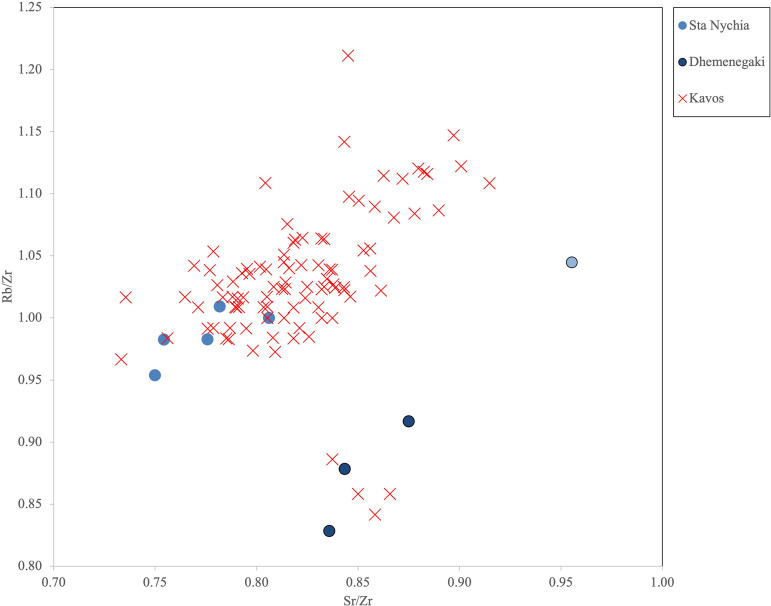
Bivariate ratio plot of Rb/Zr vs. Sr/Zr for geological samples from Dhemenegaki and Sta Nychia (Melos), plus the 100 Kavos artifacts. R. Moir. Original copyright with the authors.

The results of the elemental analysis of 103 obsidian artifacts from Kavos are presented in Table 4. An initial bivariate contents plot of strontium (Sr) vs. zirconium (Zr) clearly shows two clusters (Fig 20) with the largest group (n = 100) overlapping the products of the two Melian sources while three pieces had values that matched those of Giali A obsidian, as originally predicted by visual characterization [50]. A follow-up Rb/Zr vs. Sr/Zr ratio plot serves to discriminate the raw materials of the first 100 artifacts into two groups with 96 shown to be made of Sta Nychia obsidian and four of Dhemenegaki (Fig 21). The blade-like flake whose banding/translucency led us to record its raw material as being from ‘Nenezi Dağ or Dhemenegaki’ (K8/M/BA,1 L1, not illustrated) was shown to have been flaked from the latter obsidian.

**Table 4:** Elemental compositions of Kavos obsidian artifacts as determined by pXRF. Values expressed in ppm [parts per million]; RV = recommended values.

Artefact ID	Area	Source	Mn	Fe	Zn	Ga	Th	Rb	Sr	Y	Zr	Nb	Rb/Zr	Sr/Zr	Mn/Rb
K6/S/D1,18 L9	SDS	Sta Nychia	559	9136	36	16	14	130	102	20	125	9	1.04	0.82	4.30
K8/S/J1,27 L1	SDS	Sta Nychia	442	7763	32	16	13	117	89	17	114	9	1.03	0.78	3.78
K7/S/D2,18 L32	SDS	Sta Nychia	499	8643	40	17	11	115	92	19	117	9	0.98	0.79	4.34
K7/S/N3,28 L2	SDS	Sta Nychia	461	7765	36	15	11	114	91	16	113	10	1.01	0.81	4.04
K6/S/F3,13 L45	SDS	Sta Nychia	519	8100	23	17	12	120	94	20	119	9	1.01	0.79	4.33
K7/S/J3,68 L3	SDS	Sta Nychia	549	9243	37	16	13	123	103	19	123	11	1.00	0.84	4.46
K6/S/B4,68 L5	SDS	Sta Nychia	497	8103	37	16	12	119	99	20	117	10	1.02	0.85	4.18
K7/S/J3,88 L3	SDS	Sta Nychia	491	7870	25	16	11	115	88	16	111	9	1.04	0.79	4.27
K8/S/D4,11 L5	SDS	Sta Nychia	502	8400	24	18	11	121	93	20	123	9	0.98	0.76	4.15
K6/S/B1,4 L3	SDS	Sta Nychia	524	8504	37	16	14	124	102	19	121	10	1.02	0.84	4.23
K7/S/F3,15 L45	SDS	Sta Nychia	566	9638	38	18	14	139	108	21	127	11	1.09	0.85	4.07
K6/S/D2,2 L12	SDS	Sta Nychia	467	8446	32	16	12	123	98	18	118	9	1.04	0.83	3.80
K6/S/D1,14 L13	SDS	Sta Nychia	515	9378	37	16	14	123	101	20	125	9	0.98	0.81	4.19
K6/S/B1,3 L3	SDS	Sta Nychia	582	8167	35	12	9	107	89	17	110	9	0.97	0.81	5.44
K6/S/D1,38 L10	SDS	Sta Nychia	411	7917	29	14	13	117	90	18	113	8	1.04	0.80	3.51
K6/S/C1,4 L23	SDS	Dhemenegaki	477	9640	39	17	12	101	103	20	120	8	0.84	0.86	4.72
K6/S/C1,9 L4 SF 132	SDS	Sta Nychia	518	8430	33	16	14	123	97	20	120	9	1.03	0.81	4.21
K7/S/F2,69 L5	SDS	Sta Nychia	503	7908	36	16	11	115	90	19	116	9	0.99	0.78	4.37
K8/S/M3,28 L5	SDS	Dhemenegaki	476	9772	32	15	11	103	102	17	120	8	0.86	0.85	4.62
K7/S/F2,72 L5	SDS	Sta Nychia	566	9247	39	18	13	135	104	18	127	11	1.06	0.82	4.19
K7/S/J3,5 L4	SDS	Sta Nychia	579	8531	34	16	14	121	95	19	122	10	0.99	0.78	4.79
K6/S/C1,1 L13	SDS	Sta Nychia	565	9306	46	19	12	126	97	21	121	10	1.04	0.80	4.48
K6/S/C1,1 L3	SDS	Sta Nychia	562	9788	40	16	15	134	108	21	129	9	1.04	0.84	4.19
K8/S/F4,1 L2	SDS	Sta Nychia	451	8095	31	14	12	115	90	18	114	8	1.01	0.79	3.92
K6/S/B4,66 L5	SDS	Sta Nychia	582	9344	32	15	13	133	104	20	125	10	1.06	0.83	4.38
K7/S/C4,21 L2	SDS	Sta Nychia	612	9132	37	18	13	133	103	22	128	10	1.04	0.80	4.60
K6/S/D1,16 L9	SDS	Sta Nychia	641	10373	43	15	16	137	113	20	132	12	1.04	0.86	4.68
K7/S/F2,70 L2	SDS	Sta Nychia	594	9049	31	17	16	138	102	21	131	10	1.05	0.78	4.30
K6/S/B4,100 L6	SDS	Sta Nychia	493	9108	31	15	13	131	104	21	128	12	1.02	0.81	3.76
K8/S/D4,18 L6	SDS	Sta Nychia	502	8973	26	18	14	132	102	19	124	10	1.06	0.82	3.80
K7/S/D1,14 L32	SDS	Sta Nychia	503	8149	32	18	12	119	91	20	118	10	1.01	0.77	4.23
K6/S/B1,3 L4	SDS	Sta Nychia	515	9923	39	19	17	137	113	24	134	9	1.02	0.84	3.76
K7/S/D1,19 L31	SDS	Sta Nychia	436	8509	32	17	14	123	100	18	120	9	1.03	0.83	3.54
K8/S/K2,20 L3	SDS	Sta Nychia	492	7981	29	16	12	116	91	17	115	8	1.01	0.79	4.24
K8/S/J1,2 L1	SDS	Sta Nychia	541	8498	34	18	13	122	99	20	121	10	1.01	0.82	4.43
K7/S/F2,71 L5	SDS	Sta Nychia	543	9727	42	16	15	131	109	21	131	12	1.00	0.83	4.15
K7/S/D1,36 L32	SDS	Dhemenegaki	548	11770	49	17	11	115	116	20	134	10	0.86	0.87	4.77
K8/S/K2,22 L3	SDS	Sta Nychia	520	8369	41	16	15	123	99	20	120	8	1.03	0.83	4.23
K6/S/C1,1 L16	SDS	Sta Nychia	606	9557	36	17	11	134	105	19	126	11	1.06	0.83	4.52
K8/S/RA,1 L13	SDS	Sta Nychia	518	8306	28	16	15	121	96	20	122	10	0.99	0.79	4.28
K6/S/B1,1 L4	SDS	Sta Nychia	574	9037	31	16	13	127	103	21	125	10	1.02	0.82	4.52
K6/S/C1,1 L22	SDS	Sta Nychia	577	9353	41	17	14	130	109	22	132	10	0.98	0.83	4.44
K6/S/B1,1 L3	SDS	Sta Nychia	658	10208	46	15	14	139	109	18	125	11	1.11	0.87	4.73
K6/S/D2,1 L10	SDS	Sta Nychia	510	8697	37	16	11	123	96	20	121	10	1.02	0.79	4.15
K8/S/D4,12 L4	SDS	Sta Nychia	626	9664	45	17	13	132	107	19	125	11	1.06	0.86	4.74
K7/S/C4 L5 [IX-XII]	SDS	Sta Nychia	534	8065	35	12	13	116	88	19	120	10	0.97	0.73	4.60
K6/S/B3,14 L5	SDS	Sta Nychia	592	10672	49	19	13	142	115	22	131	11	1.08	0.88	4.17
K7/S/J3,1 L2	SDS	Sta Nychia	459	8357	38	15	13	120	93	20	118	10	1.02	0.79	3.83
K7/S/F2,32 L2	SDS	Sta Nychia	501	8010	30	16	12	116	93	18	117	9	0.99	0.79	4.32
K8/S/F4,22 L2	SDS	Sta Nychia	476	8245	30	16	11	122	94	19	120	9	1.02	0.78	3.90
K7/S/D1,13 L32	SDS	Sta Nychia	476	8286	29	15	13	113	91	18	113	9	1.00	0.81	4.21
K6/S/D3,2 L6	SDS	Sta Nychia	560	8387	36	15	14	119	95	20	121	9	0.98	0.79	4.71
K6/S/B4,101 L6	SDS	Sta Nychia	455	8274	34	18	13	120	95	18	118	10	1.02	0.81	3.79
K7/S/D3,8 L14	SDS	Sta Nychia	457	8263	30	15	10	118	94	19	117	9	1.01	0.80	3.87
K7/S/D3,2 L15	SDS	Sta Nychia	460	9211	38	19	12	131	106	19	127	10	1.03	0.83	3.51
K6/S/D2,14 L36	SDS	Sta Nychia	532	8977	50	16	14	131	102	20	129	11	1.02	0.79	4.06
K7/S/D3,1 L14	SDS	Sta Nychia	621	10918	52	18	14	136	110	22	129	11	1.05	0.85	4.57
K7/S/D3,2 L14	SDS	Sta Nychia	563	11237	84	21	12	135	104	21	123	9	1.10	0.85	4.17
K8/S/M3,1 L4	SDS	Sta Nychia	515	8261	34	16	13	118	96	20	118	9	1.00	0.81	4.36
K6/S/D3,8 L7	SDS	Sta Nychia	459	8532	29	15	14	123	97	20	118	9	1.04	0.82	3.73
K6/S/D3,26 L9	SDS	Sta Nychia	471	8337	31	14	12	119	99	20	121	8	0.98	0.82	3.96
K6/S/D3,12 L6	SDS	Sta Nychia	524	8691	36	16	12	127	104	20	124	10	1.02	0.84	4.13
K6/S/B1,2 L3	SDS	Sta Nychia	623	11064	45	17	12	138	113	20	127	10	1.09	0.89	4.51
K7/S/D1,35 L32	SDS	Dhemenegaki	548	11770	49	17	11	115	116	20	134	10	0.86	0.87	4.77
K8/S/K2,16 L3	SDS	Sta Nychia	591	11021	47	18	12	154	122	21	138	12	1.12	0.88	3.84
K6/S/B1,2 L4	SDS	Sta Nychia	588	9808	38	18	12	133	107	20	128	10	1.04	0.84	4.42
K6/S/B4,13 L7	SDS	Sta Nychia	529	8429	32	17	12	124	96	21	118	10	1.05	0.81	4.27
K7/S/C4,4 L3 [II]	SDS	Sta Nychia	672	11349	54	20	13	149	117	23	133	11	1.12	0.88	4.51
K6/S/C4,62 L5	SDS	Sta Nychia	735	12162	76	18	16	147	118	23	136	9	1.08	0.87	5.00
K8/S/D4,7 L9	SDS	Sta Nychia	502	7818	36	17	11	111	91	18	114	9	0.97	0.80	4.52
K7/S/D1,22 L31	SDS	Sta Nychia	551	9771	39	17	15	140	108	20	132	13	1.06	0.82	3.94
K8/S/M3,2 L7	SDS	Sta Nychia	541	8928	36	16	14	127	104	21	124	11	1.02	0.84	4.26
K6/S/C1,8 L3	SDS	Sta Nychia	596	9763	45	15	16	132	105	21	129	10	1.02	0.81	4.52
K7/M/AB,2 L8	Middle	Sta Nychia	732	10986	61	16	9	140	109	18	134	12	1.04	0.81	5.23
K7/M/AB,24 L3	Middle	Sta Nychia	547	9066	32	15	14	128	97	19	119	10	1.08	0.82	4.27
K7/M/AC,4 L3	Middle	Sta Nychia	661	10089	58	19	15	132	101	22	127	10	1.04	0.80	5.01
K7/M/AB,6 L8	Middle	Sta Nychia	707	9751	59	17	12	122	101	18	123	12	0.99	0.82	5.80
K8/M/AE,21 L2	Middle	Sta Nychia	583	8184	32	13	14	114	93	20	111	9	1.03	0.84	5.11
K6/S/A1, L7	SDS	Sta Nychia	662	9951	53	15	13	134	109	22	131	10	1.02	0.83	4.94
K6/S/A1, L20	SDS	Sta Nychia	816	12105	67	19	14	153	111	22	138	10	1.11	0.80	5.33
K6/S/A1, L21	SDS	Sta Nychia	658	12007	64	18	18	135	101	21	130	7	1.04	0.78	4.87
K6/S/A1, L24	SDS	Sta Nychia	567	9066	44	16	13	119	98	19	118	9	1.01	0.83	4.76
K6/S/A1, L28	SDS	Sta Nychia	606	9154	39	13	13	123	89	20	121	10	1.02	0.74	4.93
K6/S/B4,64 L5	SDS	Sta Nychia	771	12270	71	16	19	172	120	21	142	12	1.21	0.85	4.48
K6/S/C1,8 L3	SDS	Sta Nychia	596	9763	45	15	16	132	105	21	129	10	1.02	0.81	4.52
K7/S/C4,1 L5	SDS	Giali A	307	6757	25	15	16	133	58	18	104	15	1.28	0.56	2.31
K8/S/D4,12 L4	SDS	Sta Nychia	626	9664	45	17	13	132	107	19	125	11	1.06	0.86	4.74
K8/S/K2,1 L7	SDS	Giali A	284	6864	28	17	17	138	55	18	106	17	1.30	0.52	2.06
K8/S/K2,1 L11	SDS	Giali A	324	8131	30	15	19	147	68	18	108	19	1.36	0.63	2.20
K8/S/A2, L1	RS1	Sta Nychia	680	11219	39	18	11	146	113	19	131	11	1.11	0.86	4.66
K8/S/A2, L2	RS1	Sta Nychia	760	12512	69	20	14	153	113	19	134	13	1.14	0.84	4.97
K8/S/A2, L4	RS1	Sta Nychia	584	10312	59	15	13	143	118	20	129	8	1.11	0.91	4.08
K8/S/A3, L1	RS2	Sta Nychia	712	11738	58	21	12	146	115	23	134	10	1.09	0.86	4.88
K8/S/A3, L2	RS2	Sta Nychia	827	11672	55	18	16	147	118	18	131	11	1.12	0.90	5.63
K8/S/A3, L7	RS2	Sta Nychia	757	13082	77	15	14	156	122	18	136	12	1.15	0.90	4.85
K8/S/A3, L11	RS2	Sta Nychia	780	10867	55	16	18	144	114	21	140	13	1.03	0.81	5.42
K8/M/AD, L2	Middle	Sta Nychia	520	8688	35	16	11	121	91	21	119	9	1.02	0.76	4.30
K8/M/AD, L4	Middle	Sta Nychia	693	10101	40	16	12	133	102	22	125	12	1.06	0.82	5.21
K8/M/AE, L3	Middle	Sta Nychia	735	12272	42	19	13	150	118	18	134	12	1.12	0.88	4.90
K8/M/AE, L4	Middle	Sta Nychia	845	11455	56	16	11	141	108	24	137	10	1.03	0.79	5.99
K8/M/AE, L12	Middle	Sta Nychia	854	12698	83	20	15	152	120	22	136	12	1.12	0.88	5.62
K8/M/BA,1 L1	Middle	Dhemenegaki	494	9842	37	16	11	109	103	15	123	8	0.89	0.84	4.53
RGM-2		standard, avg. (n = 10)	322	12359	39	17	12	138	94	24	206	9			
RGM-2		standard, RV	273 ± 8	13010 ± 280	33 ± 2	16 ± 1	15 ± 1	147 ± 5	108 ± 5	24 ± 2	222 ± 17	9 ± 0			

### Sourcing the Dhaskalio assemblage

**Fig 22:**
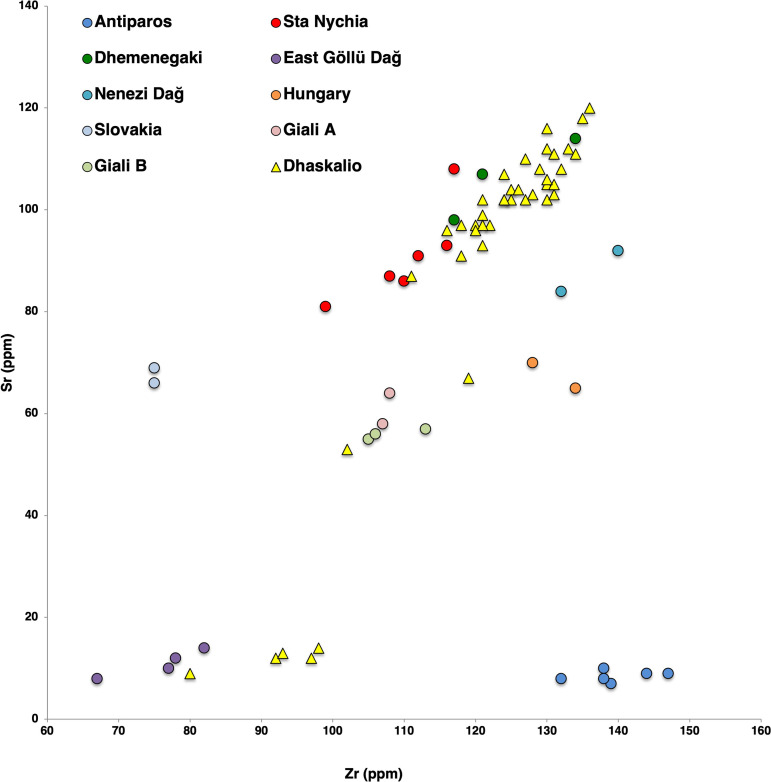
Bivariate contents plot of Zr vs. Sr for geological samples from the Aegean, Carpathians, and main central Anatolian obsidian sources, plus the 104 Dhaskalio artifacts. R. Moir. Original copyright with the authors.

**Fig 23:**
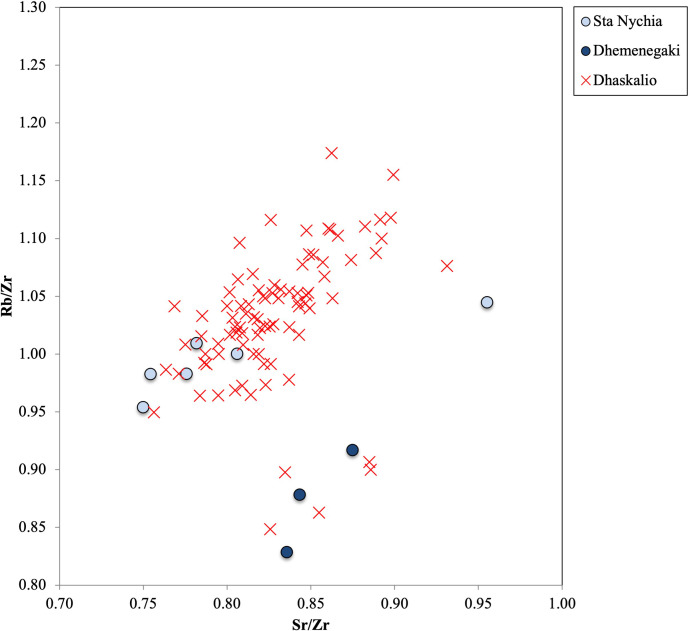
Bivariate ratio plot of Fe/Rb vs. Rb/Zr for geological samples from Dhemenegaki and Sta Nychia (Melos), plus the 104 Dhaskalio artifacts. R. Moir. Original copyright with the authors.

The results of the elemental analysis of the 104 obsidian artifacts from Dhaskalio are detailed in Table 5. In a bivariate contents plot of Zr vs. Sr, the artifacts from Dhaskalio are clearly separated into three major groups (Fig 22). The elemental values for most of the raw materials match those of Melian products (n = 88, 93%) while five have chemical signatures that broadly match those of geological samples from East Göllü Dağ (5%) with the remaining two pieces shown to derive from the Giali A source (2%). A ratio plot of Rb/Zr over Sr/Zr then distinguished the Melian raw materials (Fig 23) showing that most (n = 82/88, 93%) artifacts were made of Sta Nychia obsidian while only six artifacts were fashioned from Dhemenegaki products.

**Table 5:** Elemental compositions of Dhaskalio obsidian artifacts as determined by pXRF. Values expressed in ppm [parts per million]; RV = recommended values; * = 60 seconds.

Artefact ID	Phase	Source	Mn	Fe	Zn	Ga	Th	Rb	Sr	Y	Zr	Nb	Rb/Zr	Sr/Zr	Mn/Rb
K7/D/T II,1 L22, SF 5476	B	Sta Nychia	567	8258	40	16	11	112	89	18	113	8	0.99	0.79	5.06
K8/D/T XXX,1 L1	C	Sta Nychia	500	7779	29	17	11	108	89	19	112	8	0.96	0.79	4.63
K8/D/T V,40 L3	B	Sta Nychia	551	8470	35	18	13	117	97	19	118	9	0.99	0.82	4.71
K8/D/T VI,1 L35, SF 10641	C	Sta Nychia	568	8312	46	13	11	110	87	18	108	8	1.02	0.81	5.16
K8/D/T VI,16 L34	C	Dhemenegaki	435	10289	42	17	10	114	106	19	127	8	0.90	0.83	3.82
K8/D/T XL,5 L5	C	East Göllü Dağ	468	6083	19	20	19	184	9	25	80	25	2.30	0.11	2.54
K7/D/T II,5 L11	B	Sta Nychia	509	8621	30	16	12	125	97	20	120	9	1.04	0.81	4.07
K8/D/T VII,7 L39	C	Sta Nychia	566	9407	29	16	13	130	102	18	124	10	1.05	0.82	4.35
K7/D/T I,1 L18, SF 5951	B	Sta Nychia	534	7951	32	14	11	108	85	17	108	9	1.00	0.79	4.94
K8/D/T VI,1 L28, SF 10605	C	Sta Nychia	563	8296	39	16	13	113	89	19	112	9	1.01	0.79	4.98
K7/D/T VII,1 L8	C	Sta Nychia	435	7377	27	15	11	107	87	18	111	9	0.96	0.78	4.07
K7/D/T II,11 L3	B	Sta Nychia	530	10299	43	19	15	151	120	22	136	11	1.11	0.88	3.51
K8/D/T XXI,1 L5	C	East Göllü Dağ	708	8690	40	18	25	236	12	24	92	28	2.57	0.13	3.00
K7/D/T I,6 L20	B	East Göllü Dağ	790	9235	48	19	27	252	12	27	97	31	2.60	0.12	3.13
K8/D/T XXII,13 L3	C	Sta Nychia	585	9405	40	15	12	130	104	20	128	9	1.02	0.81	4.50
K8/D/T XX,4 L24	C	Sta Nychia	438	8840	35	17	14	127	102	21	124	10	1.02	0.82	3.45
K8/D/T XXIII,7 L14	C	Sta Nychia	655	10416	44	20	14	140	112	21	133	10	1.05	0.84	4.68
K8/D/T XXII,37 L3	C	Sta Nychia	604	9509	52	15	13	135	107	20	133	10	1.02	0.80	4.47
K8/D/T V,37 L3	B	Sta Nychia	545	9564	41	17	12	129	102	21	125	10	1.03	0.82	4.22
K7/D/T VI,1 L13	C	Sta Nychia	536	8588	32	16	11	123	97	19	121	10	1.02	0.80	4.36
K8/D/T XXIV,1 L10	C	Sta Nychia	466	8026	32	15	11	114	95	15	115	9	0.99	0.83	4.09
K7/D/T II,2 L17	B	Sta Nychia	701	10875	46	20	13	136	108	21	129	9	1.05	0.84	5.15
K8/D/T V,36 L3	B	Sta Nychia	614	11214	60	17	15	143	116	19	130	10	1.10	0.89	4.29
K8/D/T XXI,13 L3	C	Sta Nychia	648	10793	64	17	13	139	112	21	132	11	1.05	0.85	4.66
K8/D/T VII,4 L28 SF10701	C	Sta Nychia	629	8829	39	16	9	121	93	18	120	11	1.01	0.78	5.20
K8/D/T XX,1 L15	C	Sta Nychia	565	8833	38	18	13	131	102	20	127	11	1.03	0.80	4.31
K8/D/T VII,21 L34	C	Sta Nychia	507	8697	40	18	10	125	96	19	120	9	1.04	0.80	4.06
K7/D/T VII,16 L11	C	Sta Nychia	546	8959	37	15	14	130	107	20	124	10	1.05	0.86	4.20
K8/D/T V,3 L3	B	Sta Nychia	549	8417	35	18	13	116	91	19	118	9	0.98	0.77	4.73
K7/D/T I,22 L4	B	Sta Nychia	502	9286	41	16	13	132	104	22	125	11	1.06	0.83	3.80
K8/D/T XX,3 L52	C	Sta Nychia	513	8064	31	17	10	112	89	19	110	8	1.02	0.81	4.58
K7/D/II,15 L3*	B	Sta Nychia	812	11168	66	15	16	143	115	24	134	14	1.07	0.86	5.68
K7/D/T II,27 L3	B	Sta Nychia	600	10450	46	17	14	146	118	22	135	12	1.08	0.87	4.11
K8/D/T VI,3 L47	C	Sta Nychia	473	8480	29	17	11	126	102	20	121	10	1.04	0.84	3.75
K7/D/T VII,9 L5	C	Sta Nychia	566	9588	43	16	14	143	111	20	129	12	1.11	0.86	3.96
K8/D/T I,3 L26	B	Sta Nychia	573	8971	36	15	15	129	104	21	126	10	1.02	0.83	4.44
K8/D/T V,39 L3	B	Sta Nychia	538	9962	40	16	14	138	105	18	131	12	1.05	0.80	3.90
K8/D/T XXIII,1 L30	C	Sta Nychia	435	8068	34	16	12	110	93	19	113	9	0.97	0.82	3.95
K8/D/T XXII,19 L3	C	Sta Nychia	652	10622	50	18	12	139	109	19	128	10	1.09	0.85	4.69
K8/D/T II,5 L39	A	Sta Nychia	662	10390	56	15	17	144	112	21	130	12	1.11	0.86	4.60
K8/D/T XIII,3 L2*	C	Sta Nychia	852	12697	92	14	13	137	112	24	126	11	1.09	0.89	6.22
K7/D/T II, L11	B	Sta Nychia	548	9378	43	15	15	132	102	22	130	10	1.02	0.78	4.15
K8/D/T I,2 L39	B	Sta Nychia	624	10390	50	18	16	136	108	20	132	10	1.03	0.82	4.59
K8/D/T XV,11 L2 SF 11529	C	Sta Nychia	616	9140	47	16	12	123	102	20	121	9	1.02	0.84	5.01
K8/D/T XV,12 L2 SF 11529	C	Sta Nychia	753	11094	65	18	16	136	108	19	126	12	1.08	0.86	5.54
K8/D/T XX,3 L10 SF 11799	C	Sta Nychia	650	10020	50	17	14	130	103	19	124	11	1.05	0.83	5.00
K7/D/T II,1 L19	B	Sta Nychia	494	9060	40	21	15	131	103	19	128	11	1.02	0.80	3.77
K8/D/T II,1 L38	A	Sta Nychia	635	10379	43	16	12	139	112	22	133	9	1.05	0.84	4.57
K7/D/T II,1 L23	A	Sta Nychia	610	9065	45	18	14	130	103	21	131	9	0.99	0.79	4.69
K7/D/T VI,1 L4	C	Sta Nychia	485	8248	29	15	11	122	97	18	122	9	1.00	0.80	3.98
K8/D/T XXI,1 L3	C	Dhemenegaki	625	12958	50	16	11	126	124	21	140	9	0.90	0.89	4.96
K8/D/T VII,5 L24	C	Sta Nychia	615	10805	58	15	12	140	110	20	127	11	1.10	0.87	4.39
K8/D/T I,4 L44	B	Sta Nychia	492	8199	27	17	14	123	99	19	121	10	1.02	0.82	4.00
K8/D/T IV4, L7	B	East Göllü Dağ	770	8184	46	21	22	214	13	24	93	26	2.30	0.14	3.60
K8/D/T XXI,36, L3	C	East Göllü Dağ	682	8047	53	18	28	226	14	26	98	25	2.31	0.14	3.02
K7/D/T VI,1 L3	C	Sta Nychia	667	11241	57	19	17	145	111	22	131	10	1.11	0.85	4.60
K8/D/T VIII,5 L2	C	Sta Nychia	569	10292	47	17	14	139	106	22	130	10	1.07	0.82	4.09
K7/D/T VI,3 L13	C	Sta Nychia	572	10133	47	14	16	142	111	23	134	12	1.06	0.83	4.03
K8/D/T VI,1 L46	C	Dhemenegaki	495	10174	32	15	12	112	109	19	132	9	0.85	0.83	4.42
K7/D/T II,1 L25	A	Sta Nychia	544	9321	39	16	14	133	105	22	130	10	1.02	0.81	4.09
K7/D/T I,1 L6*	B	Dhemenegaki	714	12796	53	15	13	126	123	23	139	9	0.91	0.88	5.67
K7/D/T I,1, L15*	B	Sta Nychia	832	12235	57	15	16	148	116	23	143	10	1.03	0.81	5.62
K7/D/T I,1 L16*	B	Sta Nychia	828	11148	60	12	16	145	113	22	139	9	1.04	0.81	5.71
K8/D/T I,1 L22*	B	Sta Nychia	657	10458	57	14	13	139	109	19	129	11	1.08	0.84	4.73
K7/D/T I,1 L23, SF 10113*	B	Sta Nychia	578	8019	31	13	12	109	92	19	113	10	0.96	0.81	5.30
K8/D/T I,1 L35*	B	Sta Nychia	667	12201	67	16	14	141	122	22	131	10	1.08	0.93	4.73
K8/D/T II,1 L9*	B	Sta Nychia	621	9659	43	15	13	124	103	19	128	12	0.97	0.80	5.01
K7/D/T II,1 L10*	B	Sta Nychia	625	10097	45	19	12	134	104	19	127	10	1.06	0.82	4.66
K7/D/T II,2 L10*	B	Sta Nychia	639	10820	59	14	12	145	109	22	139	11	1.04	0.78	4.41
K7/D/T II,5 L11*	B	Sta Nychia	592	8780	28	17	12	107	89	16	110	9	0.97	0.81	5.53
K7/D/T II,1 L13*	B	Sta Nychia	564	11246	63	18	13	142	114	23	127	10	1.12	0.90	3.97
K7/D/T II,1 L16*	B	Sta Nychia	666	9270	32	15	12	127	102	21	126	11	1.01	0.81	5.24
K7/D/T II,1 L29*	B	Sta Nychia	669	12166	71	16	14	146	113	23	148	13	0.99	0.76	4.58
K7/D/T II,1 L30*	B	Sta Nychia	822	10372	41	14	13	132	108	19	129	12	1.02	0.84	6.23
K8/D/T II,1 L50	B	Sta Nychia	500	8075	32	18	13	119	96	18	116	9	1.03	0.83	4.20
K7/D/T II,1 L51*	B	Sta Nychia	693	10461	47	19	13	136	109	22	133	13	1.02	0.82	5.10
K7/D/T VI,3 L12	C	Giali A	429	10292	68	16	19	169	67	20	119	20	1.42	0.56	2.54
K7/D/T VI,1 L20*	C	Sta Nychia	687	8556	39	15	12	113	90	18	119	7	0.95	0.76	6.08
K8/D/T VI,1 L43*	C	Sta Nychia	626	9120	60	18	13	124	100	21	118	9	1.05	0.85	5.05
K8/D/T VI,1 L49*	C	Sta Nychia	518	8889	38	17	12	125	95	21	121	10	1.03	0.79	4.14
K8/D/T VI,1 L50*	C	Sta Nychia	536	8752	55	15	13	116	95	18	116	9	1.00	0.82	4.62
K7/D/T VII,7 L11	C	Sta Nychia	521	8375	33	16	14	126	93	20	121	8	1.04	0.77	4.13
K8/D/T VII,21 L34	C	Sta Nychia	507	8697	40	18	10	125	96	19	120	9	1.04	0.80	4.06
K8/D/T XIII,2 L8	C	Giali A	346	7192	35	17	14	138	53	17	102	16	1.35	0.52	2.51
K8/D/T XXIV,1 L2*	C	Sta Nychia	837	11358	75	18	13	132	113	23	135	10	0.98	0.84	6.34
K8/D/T XXIV,1 L3*	C	Sta Nychia	750	11138	49	19	15	144	115	22	141	10	1.02	0.82	5.21
K8/D/T XXIV,2 L3*	C	Sta Nychia	790	12064	69	22	13	151	118	21	139	11	1.09	0.85	5.23
K8/D/T XXIV,1 L7*	C	Sta Nychia	765	10906	50	15	16	140	111	21	127	12	1.10	0.87	5.46
K8/D/T XXIV,2 L8*	C	Dhemenegaki	466	10636	38	16	9	107	106	18	124	8	0.86	0.85	4.36
K/D/T XXIV,1 L13*	C	Sta Nychia	813	11104	40	16	13	147	115	20	140	10	1.05	0.82	5.53
K8/D/T XVII,1 L1*	C	Sta Nychia	769	11704	52	17	9	140	110	20	133	10	1.05	0.83	5.49
K8/D/T XVII,1 L2*	C	Sta Nychia	891	13592	59	16	12	162	119	21	138	10	1.17	0.86	5.50
K8/D/T XVII,11 L2*	C	Sta Nychia	753	10831	47	20	18	137	110	22	132	11	1.04	0.83	5.50
K8/D/T XVII,18 L2*	C	Dhemenegaki	477	10143	44	14	12	102	101	20	123	7	0.83	0.82	4.68
K8/D/T XVII,4 L5*	C	Sta Nychia	688	12641	67	19	16	157	124	24	138	10	1.14	0.90	4.38
K8/D/T XVII,5 L5*	C	Sta Nychia	759	12257	85	14	10	148	109	20	135	11	1.10	0.81	5.13
K8/D/T XX,1 L8*	C	Sta Nychia	862	11324	44	14	15	144	115	19	129	12	1.12	0.89	5.99
K8/D/T XX,1 L30*	C	Sta Nychia	557	9795	54	16	15	132	100	20	124	11	1.06	0.81	4.22
K8/D/T XX,1 L32*	C	Sta Nychia	639	11261	58	17	17	143	116	21	137	10	1.04	0.85	4.47
K8/D/T XX,1 L33*	C	Sta Nychia	679	9899	47	16	16	131	107	17	126	10	1.04	0.85	5.18
K8/D/T XX,1 L53*	C	Sta Nychia	813	12365	73	22	11	149	116	19	129	10	1.16	0.90	5.46
K8/D/T XXI,13 L3	C	Sta Nychia	648	10793	64	17	13	139	112	21	132	11	1.05	0.85	4.66
K8/D/T XXII,13 L3*	C	Sta Nychia	569	8073	45	15	8	114	93	16	114	9	1.00	0.82	4.99
K8/D/T XXII,37 L3*	C	Sta Nychia	838	11743	66	18	16	154	114	22	138	11	1.12	0.83	5.44
RGM-2		standard, avg. 120 secs (n = 5)	281	12121	38	17	12	141	96	24	203	8			
RGM-2		standard, avg. 60 secs (n = 9)	322	12569	39	17	14	140	96	23	205	9			
RGM-2		standard, RV	273 ± 8	13010 ± 280	33 ± 2	16 ± 1	15 ± 1	147 ± 5	108 ± 5	24 ± 2	222 ± 17	9 ± 0			

Based on visual inspection it was originally claimed that three pieces of obsidian from Dhaskalio were of “likely/possible” Giali origin [9]. The one lustrous, translucent, and *spherulitic* piece – which seems to be the most reliable attribute for visually determining these source products – from trench XIII level 8 was indeed proven chemically to be from Giali A while the analysis of the other two small translucent pieces *without* macroscopic spherulites showed one to be from Giali A while the other came from Göllü Dağ.

Trench XIII, Level 8: claimed to be Giali; analysis shows Giali A.
Trench I, Level 20: claimed “probably Giali, if not East Göllü Dağ”; analysis shows Göllü Dağ.
Trench VI, Level 12: claimed “probably Giali, if not East Göllü Dağ”; analysis shows Giali A.

Of the seven pieces visually assigned a “likely/possible East Göllü Dağ” origin based on their “lustrous surface, translucency, and purple-grey hue” [9], which included two of the artifacts mentioned above, the elemental analyses confirmed five as coming from Cappadocia while one piece was shown to be made of Sta Nychia obsidian and the other coming from Giali A:

Trench IV, Level 7: claimed to be East Göllü Dağ; analysis shows East Göllü Dağ.
Trench XL, Level 5: claimed to be East Göllü Dağ; analysis shows East Göllü Dağ.
Trench XX, Level 52: claimed to be East Göllü Dağ; analysis shows Sta Nychia.
Trench XXI, Level 3: claimed to be East Göllü Dağ; analysis shows East Göllü Dağ.
Trench XXI, Level 5: claimed to be East Göllü Dağ; analysis shows East Göllü Dağ.
Trench I, Level 20: claimed “probably Giali, if not East Göllü Dağ”; analysis shows Göllü Dağ.
Trench VI, Level 12: claimed “probably Giali, if not East Göllü Dağ”; analysis shows Giali A.

### Reconstructing raw material consumption traditions on Keros

The archaeometric analysis broadly confirmed our claims concerning the raw materials represented in the two Keros assemblages with Melian products dominant – comprising 97% and 94% of the Kavos and Dhaskalio study samples respectively plus smaller quantities of obsidian from the Giali A and Göllü Dağ sources (Table 6). It now requires us to integrate the sourcing results with the artifacts’ techno-typological specifics to produce a richer characterization of these two Keros assemblages and the obsidian consumption traditions represented within them.

**Table 6:** Summary results of the Keros sourcing analysis by deposit. SN = Sta Nychia, DH = Dhemenegaki, EGD = East Göllü Dağ.

Assemblage	Sample	SN	DH	Giali A	EGD
Kavos, SDS	85	79 (93%)	3 (4%)	3 (4%)	–
Kavos, Area A	7	7 (100%)	–	–	–
Kavos, Middle	11	10 (91%)	1 (9%)	–	–
Dhaskalio, Phase A	4	4 (100%)	–	–	–
Dhaskalio, Phase B	36	33 (92%)	1 (3%)	–	2 (5%)
Dhaskalio, Phase C	64	54 (84%)	5 (8%)	2 (3%)	3 (5%)
**Total**	**207**	**187 (90%)**	**10 (5%)**	**5 (2.5%)**	**5 (2.5%)**

#### The consumption of Melian obsidian.

Both the Kavos and Dhaskalio Melian obsidian assemblages embody a notable bias in raw material choice with Sta Nychia products dominant, comprising 93% and 87.5% of the characterized assemblages respectively (Table 5). When contextualized, these data provide evidence for a broader EBA tradition of Sta Nychia obsidian being the preferred Melian source material for Cycladic and Cretan communities. Most pertinent for the Kavos results, not least those from the Special Deposit South, are the sourcing data generated by Morgan [28] from the analysis of several EBA funerary assemblages from nearby Naxos and Ano Kouphonisi. In each of the five burial assemblages containing more than 10 obsidian blades, Sta Nychia obsidian comprises over two-thirds of the assemblage or three-quarters of those datasets over 20 pieces (Table 7). That the Special Deposit South blades tend to be made from the same raw material as cemetery material lends further credence to the hypothesis that at least some of this material represents disinterred grave goods from other islands that were redeposited ritually at Kavos. As to which islands and cemeteries were involved it is almost impossible to say with the obsidian assemblage (the pottery will be more informative) except for a few pieces that we believe may have originated from the rich cemetery of Aplomata associated with the trader site of Grotta on north-west Naxos. This claim is based on the presence of a few large blade fragments from this Kavos assemblage (Fig 9 [K7/S/F2,70 L2; K6/S/B4,100 L6]) whose width suggests that they were pressure flaked using the lever technique – a hypertrophic form of production restricted to the funerary arena (‘the necrolithic’), material that is best paralleled at Aplomata [101] which again was fashioned from Sta Nychia obsidian [28]. Connections between Kavos and Aplomata have already been made with reference to their distinctive grave types [51]. Furthermore, Doumas and Lambrindouakis [102] argued that Aplomata acted as one of a series of Cycladic coastal ‘depots’ where disinterred grave goods from elsewhere on these islands were first accumulated prior to being transferred to Keros.

**Table 7:** Relative proportion of Melian obsidian detailed by characterization studies in Early Bronze Age [EB] – Middle Bronze Age [MB] Aegean assemblages where the study sample is ≥ 10 artifacts. Data from [14,28,63,107] and this paper.

Site (Location)	Date	Sta Nychia	Dhemenegaki
Kavos – SDS (Keros)	Early – Late EB II	92 (96%)	4 (4%)
Dhaskalio, Phase B (Keros)	Late EB II	33 (97%)	1 (3%)
Dhaskalio, Phase C (Keros)	EB III	54 (92%)	5 (8%)
Kastri (Syros)	Late EB II	133 (90%)	15 (10%)
Agrilia (Epano Kouphonisi)	Late EB I	181 (90%)	20 (10%)
Avdheli (Naxos)	Early EB II	25 (100%)	–
Ayioi Anargyroi (Naxos)	Late EB I – Early EB II	27 (96%)	1 (4%)
Lakkoudhes (Naxos)	EBI – Late EB I	8 (67%)	4 (33%)
Tzavaris (Epano Kouphonisi)	Early EB II	56 (98%)	1 (2%)
Tsikniadhes (Naxos)	Late EB I – Early EB II	120 (75%)	40 (25%)
Phaistos (Crete)	EB I – III	10 (100%)	–
Malia (Crete)	Late EB II	16 (80%)	4 (20%)
Mochlos (Crete)	EB II	34 (87%)	5 (13%)
Mochlos (Crete)	EB III – MB I	20 (91%)	2 (9%)
Çine-Tepecik (W. Anatolia)	EBA	64 (69%)	29 (31%)
Çukuriçi Höyük (W. Anatolia)	Chalcolithic – EBA	29 (49%)	30 (51%)

While blade cores and technical pieces only formed a small proportion of the Special Deposit South material (Fig 7), several of these artifacts were included in our sourcing study (Table 2, Figs 8 and 9). Tellingly, all this material relating to the initial stages of core preparation and reduction plus the rejuvenation pieces, are made from Sta Nychia obsidian. As mentioned above, there is evidence for blade production on the edges of the Special Deposit South and some of the production debris pieces from this analysis came from such deposits (e.g., trench N3). In contrast, the three artifacts made of Dhemenegaki obsidian were all in the form of pressure blades (two crested [Fig 9]) which were probably brought to Keros ready-made – potentially in the form of disinterred grave goods. The crafting of goods intended for immediate consumption within the Special Deposits comprised another component of the ritual practices performed here [101] including not only pressure blade production but also metalworking at the nearby promontory [5]. The theatrical production and dissemination of fine pressure blades to both the living and the dead, as attested within the Agrilia (Ano Kouphonisi) and Tsikniades (Naxos) cemeteries, arguably formed part of village-based feasting ceremonies – also including the breaking and circulation of marble goods – that served to underwrite and/or terminate social relations [101,103]. One can envisage the same phenomenon being conducted on a larger and/or more exclusive scale at Kavos.

**Fig 24:**
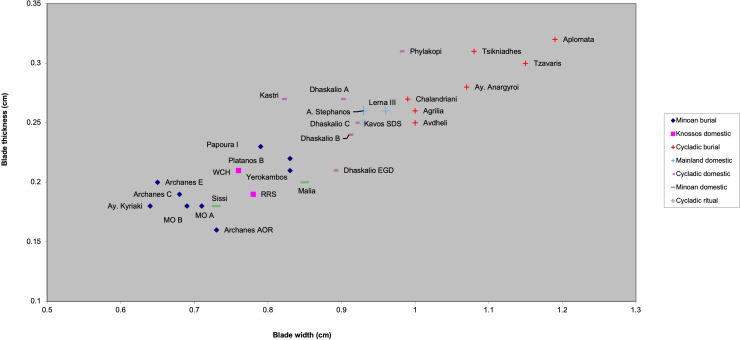
Width/thickness ratios of prismatic blades (pressure end-products) from various EBA Cycladic and Cretan assemblages, the obsidian identified visually as Melian. Data from [105], plus unpublished data from Malia and Sissi. T. Carter. Original copyright with the authors.

Turning to the Dhaskalio material, here the minority Dhemenegaki component embodies a slightly broader array of blanks than attested in the Kavos assemblage with not only end-products (one crested, and two prismatic blades) but also an exhausted core, a cortical flake, and a blade-like flake (Table 3). We also note that five of these six artifacts come from Phase C deposits, indicating a slight increase in the use of this resource over time from 3–8% of the Melian products from Phase B to C (Table 6). Although there are fewer characterized domestic Melian obsidian assemblages with which to contrast the Dhaskalio results, the one major study from Kastri on Syros [14] is very pertinent given its contemporaneity with the Phase B-C occupation and its status as another of the ‘trader sites’ [3]. While the material from Kastri lacked Giali A and East Göllü Dağ products, it similarly showed a bias towards the consumption of Sta Nychia raw materials with at least 89% of the artifacts (n = 133/149) being flaked from this obsidian, compared to the 92% (n = 33) from Dhaskalio Phase B (Table 6). Technologically the modes of core preparation, initiation, reduction, and rejuvenation are common to both Kastri and Dhaskalio while the size of the end-products appear to be much the same as evidenced by their average width and thickness of the prismatic blades (Fig 24 [the fragility of these implements means that few are recovered whole, making the comparison of lengths difficult]). On the face of it, the late EB II assemblage from nearby Panormos on Naxos is also techno-typologically directly comparable to the Dhaskalio B material [104], whereby we can tentatively suggest a pan-Cycladic domestic technical tradition at this time which may also include raw material choice alongside the specific mechanisms of pressure flaking.

When we geographically broaden the context to consider the consumption of Melian raw materials by non-Cycladic populations we begin to see distinctions in community and/or regional crafting traditions. On Crete there are three obsidian sourcing studies known to us that included EB II material with datasets from Malia and Phaistos (both subsequently palatial sites) plus the well-connected ‘gateway community’ [106] of Mochlos (Fig 2). The results of these studies once again indicate a preference for Sta Nychia products over Dhemenegaki raw materials, though the sample sizes of the first two assemblages were relatively small (Table 7). Of the 25 EB II artifacts elementally characterized from Malia [63] 20 were Melian and five were central Anatolian (discussed below) with the former material comprising 16 pieces made of Sta Nychia obsidian and four of Dhemenegaki; the high percentage of non-Aegean raw materials in this sample was due to a sampling bias towards exotica. The ten EBA artifacts analyzed from Phaistos embodied a greater chronological range (EB I – III), but all were shown to be made of Sta Nychia obsidian [107]. The final dataset from Mochlos remains unpublished (Carter pers. comm.) but consists of 39 artifacts from EB II contexts – all characterized as being made from Melian obsidian with Sta Nychia products the majority (n = 34/39, 87%) while only five pieces were made of Dhemenegaki obsidian (13%). While these Cycladic and Cretan communities (and/or those who supplied them [108]) had a common preference for using Sta Nychia obsidian to make pressure blades, significant regional distinctions remain concerning how these raw materials were consumed. In short, these populations performed different *mechanisms* of pressure flaking blade manufacture [see 109,110]. While one could point to subtle differences in core preparation and product initiation, the most obvious distinction between EB II Cycladic and Cretan pressure blade technologies concerns their respective size with those made in the Cyclades being on average longer, wider, and thicker [105] as detailed in Fig 24. While we currently lack any obsidian sourcing data for the EB II mainland or Euboea, metric data from blade assemblages of such sites as Lerna (Argolid) and Agios Stephanos (southern Peloponnese) suggest further regional differences in pressure techniques (Fig 24).

Communities in western Anatolia also procured Melian obsidian to make pressure blades and bladelets [111] though sourcing studies indicate different traditions of raw material choice amongst these populations with Dhemenegaki apparently the preferred source for Late Chalcolithic – EBA (4^th^-3^rd^ millennium cal. BC) Bakla Tepe and Liman Tepe (Fig 2) as evidenced by the analysis of 42 artifacts [29]. A little further to the south we see a more even distribution regarding the procurement of Melian raw materials by the people of Çukuriçi Höyük during the Chalcolithic to EBA [112] with 51% of the artifacts sourced to Melos coming from Dhemenegaki (n = 30 [Table 7]). Further south still we see yet more differences in community traditions with around 30% of the 100 pieces characterized from Çine-Tepecik Höyük being fashioned from Dhemenegaki obsidian [27], i.e., much more than one associates with the EBA Cycladic traditions but much less of this raw material proportionally than being accessed by those living further to the north in western Anatolia.

One final point worth bearing in mind for future studies concerns the increased consumption of Dhemenegaki obsidian at Dhaskalio in its latest period of occupation representing 8% of the Phase C Melian material analyzed (n = 5/59 [Table 6]). Notable too is that this material encompasses a greater range of the production sequence with a core and cortical flake alongside two blades and a blade-like flake (Table 3), whereas all the other artifacts made of Dhemenegaki obsidian from the earlier deposits of Kavos, Dhaskalio (Phase B), and Kastri on Syros were in the form of end-products. This reflects a not insignificant shift in crafting traditions towards the end of the EBA, though we have no Cycladic data to compare this with to see if we are dealing with a larger regional phenomenon. Only from Crete do we have a small amount of (unpublished) sourcing data of broadly contemporary date with 22 artifacts of EB III to early Middle Bronze Age [MBA] date from Mochlos, 91% of which were made of Sta Nychia obsidian (n = 20) which conversely represents an proportional increase in this raw material’s significance compared to the preceding period (Table 7) and, by extent, not following the trend documented at Dhaskalio.

#### The consumption of Giali A obsidian.

The five pieces of Giali A obsidian detailed in this study, of which three came from Kavos Special Deposit South and two from Dhaskalio, were all in the form of small (≤2.1 cm long) non-cortical chunks or flakes. This raw material outcrops massively on the eastern half of Giali, a small island in the Dodecanese ~140 km linear distance from Keros to the south-east (Fig 1). This is a visually distinctive raw material within an Aegean context and we selected all possible pieces of Giali A obsidian from the 2006−08 excavations for analysis in this study whereby it is evident that this obsidian was procured only infrequently, comprising some 0.08% (n = 3/3543) and 0.13% (n = 2/1541) of the Kavos and Dhaskalio assemblages respectively (Table 1). Another piece of spherulitic Giali A obsidian has been visually identified from the surface survey of the Kavos Promontory, an area rich in EBA metal and obsidian working debris [113] while the island-wide survey of 2012−13 generated a further five to eight pieces with the second phase of excavations at Dhaskalio (2016−18) producing four more (T. Carter, pers. obs.). Both pieces of Giali A obsidian from the 2007−08 Dhaskalio excavations came from Phase C, thus dating at least some of the material’s procurement to the EB III period – sometime between 2400–2300 cal BCE (Table 6).

The use of Giali A products by Cycladic populations is very rare. There are four flakes reported from Late Neolithic (5^th^ millennium cal BCE) from Saliagos near Antiparos (one being chemically sourced), the material again comprising a tiny proportion (0.02%) of the larger obsidian assemblage [95]. The Keros material represents only the second time artifacts of this raw material had been recognized from an EBA context in the Cyclades which is telling given how much material of this date one of us has studied from throughout the archipelago [14,66,114]. Nor is this visually distinct raw material recorded from EBA assemblages from Markiani (Amorgos) or the south-eastern Naxian site of Panormos on Naxos [104,115]. The only other instance we know of – proven via elemental analysis – is a small spherulitic nodule from the late EB I cemetery of Agrilia on nearby Ano Kouphonisi [28,66]. No Giali A material is reported from Cretan sites of this period. Where there does seem to be a tradition of using of this obsidian in the EBA is amongst communities on the neighboring island of Kos in the Dodecanese [116] and at a few sites beyond in western Anatolia, with a few pieces from EB I–II burials at Iasos [117] plus some flakes from Çine-Tepecik Höyük [27].

As discussed in the original publications, the functional significance of this handful of Giali A obsidian from Keros remains unclear. There is no indication that these small chunks and flakes comprise the waste material from knapping larger objects nor do they derive from the lapidary production of vessels or sealstones, forms of crafting we associate primarily with Middle and Late Bronze Age Crete [67,118]. If these few flakes functioned as implements a microwear study would be needed to prove it [e.g., 119]. Alternatively, the significance of Giali A obsidian to those gathering at Keros may have lay in its exoticism and/or the symbolic affordances of lustrous materials [cf. 120,121]. We note that there were other small stones with no apparent utilitarian value brought to Dhaskalio with 1954 beach pebbles – mainly from Ano Kouphonisi – deposited at the site, most of which also date to the Phase C occupation [122]. These ‘trinkets’ [cf. 123] of Giali A obsidian may also have part-articulated relations with communities to the east channeled through maritime networks through which key goods and ideas were gifted and exchanged, a thesis we expand upon in the discussion.

The mode by which these small pieces of Giali A obsidian came to be deposited at Kavos and Dhaskalio may not have been the same. Here we return to the Iasos case mentioned above, where Giali A obsidian was found in graves that are both Cycladic in style and broadly contemporary with the Kavos Special Deposit South [124,125]. We suggest that it is not inconceivable that the three pieces of this raw material from the Special Deposit South were disinterred from a cemetery in western Anatolia (Iasos and/or elsewhere) and brought for ritual deposition at Kavos in a manner analogous to that suggested for the inclusion of marble and ceramic grave goods in the special deposits [2]. Alas, such a claim does not bring us any closer to understanding the use/cultural significance of this material to the people who originally procured and buried it.

#### The consumption of Göllü Dağ obsidian.

Five artifacts from Dhaskalio were shown to have been flaked from obsidian whose chemical signatures most closely aligned with those of our geological samples from the Göllü Dağ volcanic complex in southern Cappadocia, central Anatolia, around 900 km linear distance east of Keros (Fig 1). While the values of the Göllü Dağ source products and those of the artifacts do not completely overlap on the bivariate Sr vs. Zr plot (Fig 22), we are content that the archaeological material was fashioned of obsidian from this volcano in part through reference to the other elemental values (compare to those published in [97]) and their visual characteristics [72]. This value discrepancy in values is likely due to our study including only four geological samples from Göllü Dağ, even though there are several geo-spatially, chronologically, and chemically distinct obsidian sources associated with this volcano [97]. The first reference to the circulation of Göllü Dağ obsidian at distance can be found in the early studies of Renfrew and colleagues from the 1960’s where the volcanic complex was referred to as ‘Çiftlik’ [18,19,126]. The review by Poidevin [127] suggested that there were six major outcrops that could be chemically distinguished into East and West Göllü Dağ compositional groups with the former products of greatest archaeological significance circulating throughout Anatolia, Cyprus, and south-west Asia [22,128]. Subsequent studies have discerned yet more geochemically distinct outcrops [100]. The four geological samples included in this study came from two of Göllü Dağ’s most important sources – Kömürçü and East Kayırlı [127] – whose raw materials were used at distance throughout prehistory [22,128,129]. While we cannot assign the Dhaskalio raw materials to either one of these sources with confidence, we believe that the East Kayırlı outcrops are most likely as these products are typically the purple-grey translucent variety, whereas Kömürçü products are typically an opaquer and lustrous black obsidian with a blue hue at the knapped edge [72]. We employ the term ‘East Göllü Dağ’ as the source name for the Dhaskalio material, in keeping both with the elemental data, and the terminology we have previously employed for such material in an Aegean context [32].

With all the Keros artifacts visually characterized as coming from East Göllü Dağ having been included in this study, it can be argued that that this Cappadocian material was only rarely brought to the island constituting a mere 0.3% of the obsidian from the 2007−08 Dhaskalio excavations (n = 5/1541). Thus far the extensive work on Keros [1] has only produced East Göllü Dağ obsidian from Dhaskalio with the material discussed in this paper coming from both Phase B (n = 2) and Phase C (n = 3) deposits which span ca. 2550–2250 cal BC (Table 6). Many more artifacts (40+) visually identified as being made of this Cappadocian raw material have been recorded from the more recent excavations at Dhaskalio (2016−18) – double the relative amount within the larger obsidian assemblage (~0.6%).

While small quantities of East Göllü Dağ obsidian were procured by communities along the western Anatolian coast from at least the Early Pottery Neolithic of the 7^th^ millennium cal BC [34, see also 25], this raw material – or the artifacts flaked from it – do not seem to have been accessed by Cycladic populations until the EBA. The earliest evidence for East Göllü Dağ obsidian in the Cyclades dates to the late EB I, represented by a (sourced) pressure blade from the Agrilia cemetery on neighboring Ano Kouphonisi [28,66]. To our knowledge this would have been the furthest west Cappadocian obsidian had ever been transported. We only know of one other instance of East Göllü Dağ obsidian (visually characterized) in the Bronze Age Cyclades – an exhausted pressure blade core from the Kato Kouphonisi survey; while this was a surface find, it almost certainly dates to the 3^rd^ millennium cal BC (T. Carter, pers. obs.).

Beyond the Cyclades, small quantities of Cappadocian obsidian have been reported from two other sites that should/may be contemporary with Dhaskalio. The first is the major north-coast site of Malia where a chemical analysis of EBA artifacts showed three pressure bladelets and a flake to be made of East Göllü Dağ products while a bladelet was fashioned from Nenezi Dağ obsidian, the only known example of this raw material from the insular Aegean [63]. These objects came from later EBII (Early Minoan IIB) contexts which should date the material to between the 25^th^ century to 2200 cal BC, i.e., coterminous with Dhaskalio Phases B-C [2,130,131]. The second site is Platanos B a communal tomb complex in south-central Crete (Fig 2), whose rich contents included two very fine pressure blades of a translucent purple-grey hued obsidian that almost certainly comes from East Göllü Dağ – one over 7 cm long [19,66]. The original publication also mentions a core of the same distinctive raw material [132]. The tomb has a long use spanning the EBA – early MBA (Early Minoan II – Middle Minoan II [133]), whereby the contemporaneity of East Göllü Dağ obsidian use at Platanos B and Dhaskalio cannot be proven, though the tradition of funerary consumption of fine pressure blades in a Cretan context is a primarily EB I-III phenomenon of Cycladic cultural derivation [65,66,105].

Returning our attention to the east, sourcing studies have detailed that small amounts of Cappadocian obsidian were being procured by EBA communities in the coastal region of western Anatolia. East Göllü Dağ products are evidenced at Liman Tepe [29], Çine-Tepecik Höyük [27], and Troy [134] – the latter coming from periods II-V which is contemporary with Dhaskalio Phases A-C [135] while Nenezi Dağ obsidian is known from Çukuriçi Höyük [112].

**Fig 25:**
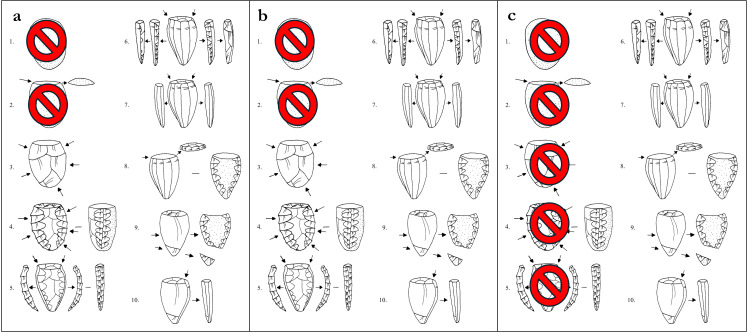
Schematic representation of the reduction sequence stages represented in the (a) Sta Nychia, (b) Dhemenegaki, and (c) East Göllü Dağ obsidian assemblages from Phase C Dhaskalio. Modified from an illustration by L. Labriola. Original copyright with the authors.

As with the Melian raw materials at Dhaskalio, East Göllü Dağ obsidian was used to manufacture fine pressure blades. Based on the five pieces detailed in this paper and the unpublished material from the 2016−18 excavations, it is evident that East Göllü Dağ obsidian was brought to the site in the form of initiated/part-reduced blade cores, i.e., in a slightly more advanced form of preparation than we see regarding how Melian obsidian arrived (Fig 25). The reduction sequence commenced with the removal of secondary series blades (with remnant cresting scars), followed by the prismatic end-products, together with small quantities of rejuvenation flakes, and intensively worked chunks that likely represent exhausted blade cores (Figs 13−14 and 16). There is also a subtle scalar distinction between the pressure blades made from East Göllü Dağ obsidian and those fashioned from Melian products (Fig 24). The former represented by a dataset of 19 blades from Phase B-C contexts (2007−08, and 2016−18 excavations), on average measure 0.89 cm wide and 0.21 cm thick compared to 0.92 cm and 0.25 cm for those made of Melian obsidian from the same periods of occupation (n = 812). This might suggest a subtly different production mechanism being employed to work the central Anatolian raw material. Finally, as with the blades crafted from Melian obsidian most of the East Göllü Dağ implements seem to have been used without further modification.

Contextually the East Göllü Dağ material from the 2007−08 excavations comes from Phase B (n = 2) and Phase C (n = 3) deposits *contra* our original claim that all bar one came from the latter period [50]. The three pieces from Phase C deposits came from the structures south of “the Hall” (trenches XXI, and XL) on the summit of Dhaskalio.

## Discussion

This study provides further evidence for the preferred consumption of Sta Nychia obsidian by populations throughout the EBA Cyclades, Crete, and parts of western Anatolia. So how do we understand these cultural traditions? Why a bias towards one Melian source over another given the raw materials’ similar properties and accessibility? In a related vein, who was responsible for procuring these raw materials? Was the habit of primarily exploiting Sta Nychia obsidian common to all communities venturing to Melos or were these choices made by a few influential intermediaries? It was once argued that the inhabitants of the prehistoric Cyclades accessed the Melian sources directly [38,62] given that obsidian working was documented in quantity throughout the archipelago. In this model, the raw material bias documented in the obsidian assemblages from Dhaskalio-Kavos, Kastri, and the Naxian cemeteries would theoretically be the result of each community having a common tradition of voyaging to the source of Sta Nychia. Alternatively, it may have been the case that the most socio-economically significant maritime voyages within the Cyclades were controlled by the few communities capable of crewing paddled longboats, i.e., the ‘trader sites’ [78,79]. In this second scenario the choice as to which obsidian the hamlet-dwelling populations of the islands received was made by the non-local specialist longboaters who procured and transported it from Sta Nychia. In the case of Kastri, one of these ‘trader sites’, we thus argued that the bias towards Sta Nychia obsidian was the result of that population’s decision making or at least those members of the community involved in such overseas ventures [14]. On the face of it we should be able to forward the same argument for Keros [3,78] with members of Dhaskalio’s longboat expeditions directly targeting Sta Nychia raw materials. However, with many seeing Keros as a socio-religious attractor site/sanctuary [1,2,103], it follows that much of the material brought to Kavos and Dhaskalio was likely transported to the island by non-locals rather than the residents themselves. As such, the dominance of Sta Nychia obsidian within the Dhaskalio-Kavos assemblages cannot be viewed as solely the result of local agency but instead reflects cultural habits operating at a pan-Cycladic level at the very least.

The above statements pertain primarily to the EB II period as represented by the Kavos Special Deposit South material and that from Dhaskalio Phase A-B. Much less is understood about the broader regional traditions and modes of Melian obsidian procurement in the EB III period of Dhaskalio C. This latter part of the 3^rd^ millennium cal. BC saw a significant reconfiguration of cultural lifeways in the Cyclades, including the abandonment of many sites and likely fragmentation of social networks [131,136]. Importantly, this is also the period to which the ‘great obsidian workshop’ of Phylakopi belongs [62]. The huge quantities of blade cores and associated manufacturing debris recovered from this deposit indicate that the main community of Melos (Fig 2) was working obsidian at a level almost certainly above and beyond the needs of the immediate population with quantities of the pre-formed nuclei and/or blades likely then exchanged with other communities on the island and overseas [68]. The occupants/craftworkers of Dhaskalio Phase C might conceivably been some of the recipients of Phylakopi’s surplus products; a future comparison of the raw material ratios represented in these assemblages would be informative in that respect.

As to why Sta Nychia obsidian was the preferred Melian raw material for so many communities in the EBA (or for the intermediaries who provisioned them), we have previously suggested that maritime technology may have influenced matters [14]. The protected coastline closest to Sta Nychia was arguably a more attractive location for anyone arriving by paddled longboat whereas the clifftop outcrops of Dhemenegaki overlooking exposed waters may have been less appealing. That said, with other contemporary and earlier communities in Crete and western Anatolia preferring Dhemenegaki obsidian [29,107], the ‘safe-harbor-access’ model can only be viewed as part of the story. Ultimately, these distinctions in raw material choice, particularly when dealing with broadly contemporary later EB II sites, embody the different social networks and factions responsible for the overseas circulation of Melian obsidian and these peoples’ material preferences and value regimes.

We turn now to non-Melian obsidian from Keros. What is the significance of this handful of material, the small flakes and chunks from Giali A in the Dodecanese, and the pressure blade related material from the central Anatolian source of East Göllü Dağ? Given the small quantity, visual distinctiveness, and distant origin of the raw materials these artifacts’ value may have lain more in their non-utilitarian affordances [cf. 137,138] than their functional capabilities. Both datasets are also emblematic of cultural connections to the east, albeit in potentially distinct fashions. Concerning Giali A obsidian, we are tempted to emphasize that the first appearance of this raw material in the Bronze Age Cyclades dates to the late EB I. This is the period associated with the first major wave of Cycladic driven ‘internationalism’ in the Aegean when an array of material culture and attendant social practices were exported to and/or emulated by communities on the surrounding coastlines [44,66,83,108,139,140]. Connections with littoral communities of north-central and eastern Crete were particularly strong at this time, perhaps even representing ‘Cycladic colonies’ [141,142]. Given the currents and winds associated with the open water between the southern Cyclades (Thera) and northern Crete, it has been suggested that a voyage involving paddled longboats would likely have taken a circular route rather than a simple back-and-forth. The return leg would have taken an anticlockwise course from the tip of eastern Crete via Kasos into Dodecanese tramping northwards to Kos and/or Kalymnos before then paddling westwards back into the Cyclades via Amorgos [143]. Given that Giali is situated on this maritime course, one could envisage a few small pieces of obsidian being collected en route this distinctive white spotted material perhaps affording the mariners a memento of their distant voyage and/or protection cf. [144,145].

While the above scenario suggests a mode of low-level direct procurement of Giali A obsidian by Cycladic mariners, the procurement of East Göllü Dağ obsidian almost certainly involved Anatolian populations moving the raw material and/or preformed cores westwards to the coast. As noted above, East Göllü Dağ obsidian has been documented at a handful of western Anatolian EBA sites: Çine-Tepecik Höyük, Liman Tepe, and Troy [27,29,134]- albeit in such small quantities that we hesitate to suggest that these were the suppliers of Dhaskalio. Instead we believe that this obsidian was moving in tandem with other eastern materials desired by Cycladic factions, not least metals.

The late EB II phase in the Cyclades (Dhaskalio B) is associated with the appearance of non-local material culture and technologies whose origins are generally agreed to be Anatolian or further to the east. These included new feasting kits, the earliest use of the potters’ wheel, new forms of combat technology, and innovations in metalworking [146–148], including evidence for the use of non-Cycladic copper [149]. While some argued that these novel features were the result of population incursions [149–151], we believe that these changes were due to local populations adopting new modes of social distinction part-achieved by establishing closer socio-economic relations with western Anatolian communities e.g., [3,152]. These coastal communities such as Liman Tepe, Bakla Tepe, Iasos, and Miletus *inter alia*, all of whom evidence connections with the Cyclades through their use of Melian obsidian [111,117,153], acted as portals to a much larger international realm, the so-called ‘Anatolian Trade Network’ that reached from Mesopotamia to western Anatolia, extending to Cyprus, the Cyclades, Greek mainland, and into the Balkans [151,154].

Eastern materials and influences at Dhaskalio Phase B-C are present in the ceramic assemblage [6] and some of the weapon types [5] with a couple of Indus-style carnelian beads likely having travelled the furthest [155]. The most socially significant goods procured by Aegean communities through the Anatolian Trade Network [151] were likely metals including copper, gold, and tin. Even though copper is native to the Cyclades, with known EBA exploitation of sources on Kythnos [156] and Seriphos [157], there is evidence for the procurement of Anatolian copper by Cycladic communities from late EB II [149]. Gold is extremely rare in the EBA Cyclades, yet Dhaskalio has produced a few objects with one late EB II/ Phase B bead that has good parallels from Troy IIg [158]. This northwest Anatolian connection is significant with a recent characterization study of late EB II goldwork suggesting that this was a core area in the dissemination of raw materials and crafting traditions with jewellery from Troy IIg and Poliochni (Lemnos) showing common techniques and resources as those documented from the Royal Graves of Ur in southern Mesopotamia [159]. Tin is the other new resource procured from the east by EBA Cycladic populations, the necessary constituent of bronze, an alloy that not only accords end-products a greater strength but also produces a different colour to other copper alloys, a seemingly important characteristic given that some of the earliest tin-bronzes of the Aegean were ornaments rather than utilitarian tools [160,161]. From Keros there are thus far only two published tin bronze objects including an axe from a small hoard found in the Phase C Hall [5].

We see the circulation of Cappadocian obsidian and Anatolian/Asian metals as inextricably related in the Bronze Age perhaps most strongly connected to the westward movement of tin. During the EBA this may have involved products from the Kestel mine (Fig 1) in the central Taurus [162] with texts from Ebla in Syria detailing the movement of Anatolian tin over distance (in the direction of southern Mesopotamia) from at least the 24^th^ century BC [163]. Recent lead isotope analyses of bronzes from the palatial EBA (2500–2200 cal BC) site of Kültepe in central Cappadocia indicate that the tin used at this time was local rather than of central Asian origin, as was the norm from the MBA onwards [164]. The connection between the circulation of Anatolian tin and Cappadocian obsidian part lays in the proximity of these sources to one another with the Kestel mine only 60 km southeast of Göllü Dağ while the major (palatial) political and economic hub of Kültepe [165] lays ~110 km to the northeast of the obsidian sources (Fig 1). We suggest that the westward flow of tin and obsidian via the EBA Anatolian Trade Network [151] comprised an earlier and smaller-scale iteration of what subsequently became a highly organised supra-regional trade network. In the MBA metals and textiles were circulated in bulk via overland caravans, moving tin from central Asia via southern Mesopotamia northwards into the kingdoms of central Anatolia via the so-called Old Assyrian trade network [166–168] and thereafter westwards via terrestrial then riverine routes to the Aegean. During the MBA we again see a small amount of East Göllü Dağ obsidian circulating in the Aegean, albeit restricted to palatial and other elite contexts in Protopalatial Crete which may reflect the increased value this resource seems to have been accorded back in Cappadocia, through it being fashioned into ornaments and vessels and stored in bulk in King Anitta’s palace [32]. Just as the value of tin may have part-rested on the novel colour bronzes imparted, the translucent purple-grey products of Kayırlı (East Göllü Dağ) were also very distinctive, not least amongst those using Melian raw materials (Fig 18). Thus, while the desire for eastern metals was the prime driver for Cycladic factions negotiating relations with Anatolian gateway communities, it might be a mistake to see the presence of small quantities of East Göllü Dağ obsidian as epi phenomenal. It is also worth noting that Dhaskalio is only the second place in the Cyclades where we see a co-occurrence of obsidian from East Göllü Dağ, Giali A and Melos (after late EB I Agrilia, Ano Kouphonisi [28]), the next occurrences being elite contexts in Palatial Crete such as *Quartier Mu*, Malia [32] and, most evocatively, the Vat Room deposits in the Central Palace Sanctuary at Knossos where their different colours, lustrousness, and translucency may have been of particular import alongside an array of other brilliant media such as gold, rock crystal, and faience [169].

## Conclusions

The elemental characterization of the 207 obsidian artifacts EB II – III Keros forms part of a recent trend towards the interrogation of large datasets in Mediterranean obsidian sourcing studies [e.g., 87,170,171 *inter alia*] in contrast to earlier work whose conclusions often rested upon the analysis of only a few artifacts per site [19,126,172,173 *inter alia*]. These larger scale analyses have largely been facilitated by the introduction of rapid, non-destructive, and relatively cheap XRF instrumentation [90,174], not least portable or hand-held techniques that enable the analyst to work on entire assemblages in cultural heritage repositories rather than exporting a sub-sample of the dataset to lab-based instruments. While this study represents the largest dataset of obsidian artifacts to have been fully published from the prehistoric Aegean, the material still comprises only ~2.5% and ~6.5–7% of the larger Kavos Special Deposit South and Dhaskalio obsidian assemblages respectively (Table 1). Constraints of time and money determined the sample size with the aim being to significantly increase the number in future analyses.

This paper had three main research questions in mind: (1) to reconstruct the socio-economic networks that coalesced at Keros, (2) to map distinct cultural traditions within the EBA Aegean, and (3) to contribute to a long-term history of Aegean obsidian source exploitation. Concerning the first issue, our results indicate that the Kavos and Dhaskalio obsidian assemblages embody a significantly larger and more complex web of connectivity than reflected in most other datasets of the EBA insular Aegean. The affordances of the island and/or the influence of those who gathered/dwelled on Keros resulted in people, goods, and practices being drawn here from near and afar, the handfuls of Cappadocian obsidian and Indus carnelian testimony to the supra-regional networks that coalesced here. The only other sites we can point to that have produced broadly comparable assemblages are those from the cemetery of Agrilia on neighboring Ano Kouphonisi [28,66], which in tandem with the rich material from the nearby Tsavaris plot [175,176] may come to represent an earlier iteration of Keros. The other site is late EB II Malia in north-central Crete [63], another major regional gathering place with architectural features and social practices that prefigure those of the MBA palace [177]. While the flow of Giali A and East Göllü Dağ obsidian westwards to Keros was entangled within the movement of perhaps more significant resources such as metals, these raw materials/artifacts were capable of encoding and transmitting social information through reference to their rarity and highly distinctive visual properties [cf. 178] constituting desirable media for initiating and cementing social relations.

This study has also made a significant contribution to our mapping of EBA (late EB II) communities of practice via a multi-faceted characterization of obsidian consumption traditions amongst southern Aegean populations. If one focused on raw material choice alone then a case can be made that Cycladic, Cretan, and certain western Anatolian populations all preferred Sta Nychia obsidian – a practice that one might propose represents common cultural traditions (Table 7). However, by locating these sourcing data within a broader *chaîne opératoire* analytical framework [179] that considers the various cultural choices involved in an artefact’s life, i.e., not only regarding raw material procurement but also those involved in its technical-stylistic transformation, then it is possible to elicit far more meaningful patterns from the archaeological record. Such an integrated approach has enabled us to discriminate Cycladic, Cretan, and western Anatolian communities of practice within the overarching framework of ‘those populations preferring Sta Nychia obsidian’ (Fig 24). On the face of it one might worry that this study has simply reinvented the region’s long established ‘Cycladic/ Minoan/ Anatolian’ cultural-historical wheel [81], yet we point to the distinctions characterization studies have made between EBA populations *within* western Anatolia: Liman Tepe and Balka Tepe preferring Dhemenegaki products with Sta Nychia dominant at Çine-Tepecik Höyük. In time, we expect further sub-regional differences in EBA obsidian consumption traditions to emerge across the southern Aegean with the potential for site-specific communities of practice, as suggested with reference to late EB II obsidian working at Mochlos [180].

Concerning our third aim to further detail the long-term history of Aegean obsidian source exploitation, the Keros study provides yet further evidence that the two Melian sources had distinct histories of use with many southern Aegean communities preferring to use Sta Nychia products throughout the Bronze Age. This claim is based on the elemental analyses of more than 14 assemblages and >1000 artefacts from Crete, the Cyclades, and western Anatolia (Table 7); the major gap in our knowledge concerns the practices of mainland and Euboean communities, several of whom were culturally connected with the EBA Cyclades [139,181,182]. As the number of sourcing studies increases, we need to consider the data at a more detailed chronological, and regional level. To that end the Dhaskalio results suggest some interesting developments from Phase B to C with a relative increase in the proportion of Dhemenegaki obsidian within the Melian products from 3% – 8% together with most of the East Göllü Dağ obsidian. Phase C also saw the introduction of a new harvesting technology with the appearance of glossed sickle blades of chert and radiolarite [9], tools that may have been procured from the Saronic Gulf, Anatolia, and/or the Levant [see 68].

Obsidian sourcing constitutes a powerful means of reconstructing interaction and engaging with issues pertaining to cultural identity and value regimes from the community to supra-regional scale. We have long argued [10] that such analyses’ interpretative potential can achieved by undertaking the kind of multi-faceted ‘thick description’ [cf. 183] approach represented in this paper. The next step is to consider consumption [cf. 184] more holistically through reference not only to traditions of tool use and discard, but also how obsidian working and tool circulation were entangled in a web of other practices. Thus, the next stage of work at Keros – on the Dhaskalio material specifically – should be a more contextualised obsidian characterization study, one that accords more attention to the spatial configuration of these data and the interrelationship of working and using these different Aegean and Anatolian raw materials with other forms of crafting (not least metalworking), storage, feasting, and the use of other exotic media such as the goldwork and carnelian. With the Keros project producing granular contextual data [185,186], such characterization/consumption studies are eminently achievable though they risk becoming more cumbersome and denser in the process. A stacked approach may thus be beneficial, one that initially dives deep, for example, into crafting practices at Dhaskalio synchronically and diachronically, followed by a secondary paper that situates the results from the first study in broader regional and supra-regional contexts. A detailed site-specific analysis may also pay dividends regarding the ability to discern and map micro-differences production across the site and/or through time through reference to raw materials, technical strategies (mechanisms of core preparation and rejuvenation), and blade size. Should such work detail the synchronic presence of subtly different practices, perhaps reflective of what Redman [187] referred to as the recognition of distinct ‘analytical individual’, it could be taken as corroborative evidence for the hypothesis that craftworking at Dhaskalio was performed by individuals from different islands who gathered cyclically at Keros to perform their trades rather than a related perennially resident social group [1]. That said, we have little idea as how homogeneous or heterogeneous such practices would be within a more typical village community of the period as for example represented by Kastri on Syros or Markiani on Ios [80,188].

While the exceptional character of Keros has long been acknowledged, the complexity of the activities that took place on Kavos and Dhaskalio is only now becoming clear through such work as this obsidian characterization study. Keros was a location where multiple socio-economic networks coalesced with a flow of people, goods, and innovative practices being attracted to Dhaskalio and Kavos in the form of social obligation and gifting, pilgrimage and dedication, with traders, raiders, and craftspeople paddling to the island for spring-through-autumn gatherings [cf. 143]. This heady congregation of people, resources, and technical know-how formed a key mode of social reproduction within Cycladic society (if not a larger scale) with aspects of social practice performed at Keros, not least the mortuary/commemorative rituals on Kavos and the commensal gatherings on Dhaskalio providing recognised spaces for the initiation, maintenance, celebration, or termination of social relations [cf. 189]. In a related vein, Keros was clearly an arena of transformation, attracting various non-local resources – not least metals and obsidian – to Dhaskalio with a community of craftworkers then producing an array of multi-media weapons, tools, and adornments – many of which were then put back into wider circulation overseas, binding people to one another and to Keros. These resources arrived in different forms – ore, metal, part-worked nodules, preformed, and part-reduced cores – drawn from multiple sources, some from vast distances, as with the Cappadocian obsidian, testimony to Keros’ power of attraction and its status as a regional religious and proto-urban centre with “exceptional reach” [1].
